# Novel Autoantigens Associated with Lupus Nephritis

**DOI:** 10.1371/journal.pone.0126564

**Published:** 2015-06-22

**Authors:** Sachiko Onishi, Endy Adnan, Jun Ishizaki, Tatsuhiko Miyazaki, Yuki Tanaka, Takuya Matsumoto, Koichiro Suemori, Masachika Shudou, Takafumi Okura, Hiroyuki Takeda, Tatsuya Sawasaki, Masaki Yasukawa, Hitoshi Hasegawa

**Affiliations:** 1 Department of Hematology, Clinical Immunology and Infectious Diseases, Ehime University Graduate School of Medicine, Ehime, Japan; 2 Department of Pathology, Gifu University Hospital, Gifu, Japan; 3 Integrated Center for Sciences, Ehime University, Ehime, Japan; 4 Department of Cardiology, Pulmonology, Hypertension and Nephrology, Ehime University Graduate School of Medicine, Ehime, Japan; 5 Proteo-Science Center, Ehime University, Ehime, Japan; INSERM-Université Paris-Sud, FRANCE

## Abstract

Systemic lupus erythematosus (SLE) is characterized by production of a variety of autoantibodies. Although anti-double-stranded DNA (anti-dsDNA) antibodies contribute to the pathogenesis of lupus nephritis (LN), they are not sufficient for diagnosis and evaluation of disease activity. To obtain other autoantibodies associated with LN, we screened autoantigens reacting with the sera of LN patients by using an N-terminal biotinylated protein library created from a wheat cell-free protein production system. We screened 17 proteins that showed higher positive signals in the active phase than in the inactive phase of SLE, and higher positive signals in the serum of SLE patient with nephritis than in that of patient without nephritis. Of these, two LN-associated autoantigens, ribosomal RNA-processing protein 8 (RRP8) and spermatid nuclear transition protein 1 (TNP1) were identified by immunoprecipitation and immunofluorescence of renal tissues. Circulating anti-RRP8 and anti-TNP1 autoantibodies were recognized and deposited as an immune complex (IC) in glomeruli. IC was deposited preferentially in glomeruli rather than in other organs in C57BL/6 mice injected with RRP8 or TNP1. ELISA analysis of sera from patients with various rheumatic diseases demonstrated reactivity for RRP8 and TNP1 in 20% and 14.7% of SLE patients, respectively, whereas there was little or no reactivity in patients with other rheumatic diseases. Among SLE patients, 63.6% and 45.5% of those with LN were positive for anti-RRP8 and anti-TNP1 antibodies, compared with 12.5% and 9.4% of SLE patients without nephritis, respectively. Both proteins are cationic, and their respective antibodies did not cross-react with dsDNA. These proteins released from apoptotic cells form ICs with each autoantibody, and their ICs may become trapped at anionic sites in the glomerular basement membrane, leading to deposition in glomeruli. These autoantibodies may be useful for prediction of LN in subsets of SLE patients who are negative for anti-dsDNA antibodies.

## Introduction

Systemic lupus erythematosus (SLE) is an autoimmune disease characterized by production of a wide variety of autoantibodies directed at various self molecules present in the nucleus, cytoplasm and cell surface [1–3]. Lupus nephritis (LN) is one of the most serious manifestations of SLE and is associated with significant morbidity and mortality [4, 5]. Renal biopsies demonstrate the presence of immune complex (IC) deposits in the renal glomeruli of patients with LN. The formation of glomerular immune deposits is a major event that initiates glomerular injury and subsequent loss of renal function. However, the mechanisms leading to the formation of immune deposits and the development of renal lesions are not yet fully resolved. In addition, the targets of pathogenic antibodies in glomeruli are also not well defined.

Anti-double-stranded DNA (anti-dsDNA) antibodies are involved in the pathogenesis of LN, and their titer is correlated with disease activity [4–6]. However, the correlation between anti-dsDNA antibodies and LN is clinically imprecise, as some patients with active nephritis are negative for the antibodies, whereas some patients showing a persistently high antibody titer may not have renal involvement [7]. In addition, deposition of anti-dsDNA antibodies in glomeruli in LN accounts for no more than 10–20% of eluted IgG overall, indicating that many antibodies other than anti-dsDNA antibodies may be associated with the pathogenesis of LN [8]. To date, some autoantibodies such as anti-C1q, anti-nucleosome, anti-Sm, anti-α-actinin, anti-α-enolase, anti-annexin II, anti-annexin AI, and anti-ribosomal P protein, have been reported in patients with LN [9–28]. However, these autoantibodies are not sufficiently sensitive or specific for prediction of LN or renal flares.

In the present study, to obtain clinical markers for the diagnosis and evaluation of disease activity in LN patients, we screened autoantigens reactive with serum antibodies using an N-terminal biotinylated protein library (BPL) produced using a wheat cell-free protein production system, and a commercially available luminescence system (BPL-based screening method) [29, 30]. The BPL-based screening method has a number of excellent characteristics, including 1) utilization of a high-throughput and genome-wide protein expression system, 2) specific protein labeling for assay using unpurified protein samples, and 3) a high-throughput system for detection of properly folded antigen. Therefore, this method is suitable for identification of autoantigen proteins reacting with antibodies that recognize folded proteins, rather than denatured or unfolded forms. In addition, since this system is fully automated, large numbers of autoantigens can be screened easily and rapidly. From this system and subsequent immunoprecipitation analysis, we found two new candidates of LN-associated autoantigens, ribosomal RNA-processing protein 8 (RRP8) [31] and spermatid nuclear transition protein 1 (TNP1) [32]. Human RRP8 is a cationic protein consisting of 456 amino acids (a.a.), localized mainly in the nucleolus. RRP8 is an essential component of the eNoSC (energy-dependent nucleolar silencing) complex, which mediates silencing of ribosomal DNA (rDNA) in response to intracellular energy status and acts by recruiting histone-modifying enzymes [33–35]. On the other hand, human TNP1 is also a cationic protein consisting of 55 a.a. TNP1 is an abundantly expressed basic protein in the sperm nucleus and participates in chromatin condensation by replacing somatic-type histones with protamines during spermiogenesis [36]. Here we describe the identification and characterization of RRP8 and TNP1 as LN-associated autoantigens.

## Materials and Methods

### Ethics statement

Approval for this study was obtained from the Institutional Review Board of Ehime University Hospital. Paraffin-embedded, formalin-fixed renal sections obtained at autopsy from LN patients were also employed. Written informed consent was obtained from each patient, or from the family in the case of autopsied patients. All *in vivo* mouse experiments were approved by the Ehime University animal care committee.

### Patients

Patients with the following diseases were analyzed in this study: SLE (n = 75), dermatomyositis/polymyositis (DM/PM) (n = 30), systemic sclerosis (SSc) (n = 16), mixed connective tissue disease (MCTD) (n = 15), rheumatoid arthritis (RA) (n = 55), Sjögren syndrome (SS) (n = 12), Behçet’s disease (BD) (n = 22), and ANCA-associated vasculitis (AAV) (n = 13). These diseases were diagnosed according to the ACR (American College of Rheumatology), EULAR (The European League Against Rheumatism), and international criteria [37–44]. In addition, 41 healthy individuals were also analyzed. Disease activity in patients with SLE was determined by calculation of the Systemic Lupus Erythematosus Disease Activity Index (SLEDAI), based on clinical findings in the two weeks prior to sample collection [45]. The active and inactive phases of LN were defined as 4≤ and 0 of the renal SLEDAI score, respectively. The diagnosis of LN was verified by renal biopsy in 7 patients, and based solely on the SLEDAI definition in 4 patients who were not obtained informed consent of renal biopsy. Information on the characteristics of 11 LN patients is shown in S1 Table.

Serum samples were frozen at -80°C until use. Formalin-fixed paraffin-embedded renal sections obtained from LN patients by kidney biopsy or autopsy were used in this study, as frozen renal sections had not been stocked.

### Preparation of a N-terminally biotinylated protein library using a wheat germ cell-free protein synthesis system

A BPL was prepared using wheat germ extract (ENDEXT kits, CellFree Sciences, Matsuyama, Japan) as described previously [29]. Briefly, we selected proteins encoded by genes in the human autoimmune susceptibility loci associated with SLE, RA, etc. [46–48], as well as proteins classified as “membrane proteins” and “proteins in the extracellular space” in the Gene Ontology database, as tentative autoantigens. A total of 2,296 genes encoding these target proteins were translated from a human full-length cDNA resource (mammalian gene collection, MGC clone, DANAFORM, Tokyo, Japan). From the plasmid-inserted cDNA, transcription templates were synthesized by split-primer polymerase chain reaction (PCR). The promoter and transcription enhancer sequences for SP6 RNA polymerase, and the coding sequence of the biotin ligase recognition peptide, were inserted into the contructs.

Cell-free construction of BPL was based on the previously described bilayer diffusion system in which 1 μL (50 ng) of crude cell-free-expressed biotin ligase was added to the translation layer and 500 nM D-biotin (Nacalai Tesque, Kyoto, Japan) was added to both the translation and substrate layers [29]. *In vitro* transcription and cell-free protein synthesis for BPL were performed with the GenDecoder 1000 robotic synthesizer (CellFree Sciences).

### Antigen screening with the AlphaScreen assay

The AlphaScreen assay (PerkinElmer Life and Analytical Sciences, Boston, MA, USA) was carried out using a 384-well Optiwell microtiter plate as described previously [30]. Briefly, 1 μL of the translation solution containing biotinylated proteins was added to a well with 14 μL of buffer containing 0.025 μL of serum. The mixture solution at a final concentration of 100 mM Tris-HCl (pH 8.0), 0.01% (v/v) Tween 20 and 0.1% (w/v) bovine serum albumin (BSA), was incubated at 26°C for 30 min. Then, 10 μL of a mixture of streptavidin-coated donor beads and protein G-conjugated acceptor beads (PerkinElmer Life and Analytical Sciences) was added, and incubated at 26°C for 1 h in a dark box. The final concentration of the beads was 12 μg/mL per well. Fluorescence emission at 520–620 nm was measured on the EnVision plate reader (PerkinElmer Life and Analytical Sciences), and the resulting data were analyzed with the AlphaScreen detection program. All repetitive mechanical procedures were performed on a Biomek FX robotic workstation (Beckman Coulter, Fullerton, CA, USA). Translation solution reacted without template RNA served as a negative control. Signals in the negative control well were regarded as noise, and the signal/noise ratio was calculated.

### Immunoprecipitation and immunoblotting

Immunoprecipitation analysis using biotinylated proteins was carried out as described previously with some modifications [29]. Briefly, 50 μL of translation mixture containing biotinylated proteins was incubated with 150 μL of IP buffer [phosphate-buffered saline (PBS) with 0.1% (w/v) BSA and 0.15% (v/v) Tween 20] and 20 μL of serum overnight at 4°C. Immobilized Protein G Sepharose (20 μL of 50% slurry, Protein G Sepharose 4 Fast Flow, GE Healthcare, Buckinghamshire, UK) was added to each sample and incubated for 1 h at 4°C. After three washes with IP buffer, samples were boiled for 5 min in sodium dodecyl sulfate (SDS) sample buffer (Bio-Rad Lab, Hercules, CA, USA). Each sample was fractionated on SDS-12% polyacrylamide gels and transferred onto PVDF membranes (Merck Millipore, Billerica, MA, USA). After blocking with 5% (w/v) skim milk in PBS overnight at 4°C, the membranes were incubated with streptavidin-horseradish peroxidase (HRP) (Mabtech AB, Nacka Strand, Sweden) for 1 h at room temperature. After washing three times in PBS containing 0.1% (v/v) Tween 20 (PBST), the biotinylated proteins on the membrane were detected by ECL detection reagents (GE Healthcare) using an ImageQuant LAS4000 (GE Healthcare).

### Purification of recombinant proteins in a cell-free system

RRP8 and TNP1 were prepared in a cell-free protein synthesis system using wheat germ ribosomal RNA [49]. A full-length cDNAs of RRP8 (human) and TNP1 (human and mouse) were obtained from DANAFORM. A full-length cDNA of mouse RRP8 was prepared from spleens of C56BL/6 mice with reverse transcriptase polymerase chain reaction (RT-PCR). The cDNA of RRP8 or TNP1 was inserted into a pEUE01-His-TEV-N2 expression vector containing a His tag region (CellFree Sciences). Both proteins were automatically synthesized by the Robotic Protein Synthesizer Protemist DT II (CellFree Sciences). Briefly, 250 μL of transcription mixture containing 25 μg of the plasmid DNA, 80 mM HEPES-KOH (pH 7.8), 16 mM magnesium acetate, 2 mM spermidine, 10 mM dithiothreitol, 2.5 mM each nucleoside triphosphate, 250 U of SP6 RNA polymerase (Promega, Madison, WI, USA) and 250 U of RNasin (Promega) was incubated for 6 h at 37°C. Then, the transcription solution containing transcribed mRNA was mixed with 250 μL of wheat germ extract WEPRO7240H (CellFree Sciences) supplemented with 1 μL of 20 mg/mL creatine kinase in a single well of a six-well plate. The substrate mix [30 mM HEPES-KOH (pH 7.8), 100 mM potassium acetate, 2.7 mM magnesium acetate, 0.4 mM spermidine, 2.5 mM dithiothreitol, 0.3 mM amino acid mix, 1.2 mM ATP, 0.25 mM GTP, and 16 mM creatine phosphate] in a volume of 5.5 mL was added on top of the translation mix and then incubated at 17°C for 20 h. The reaction mixture was then applied on Ni Sepharose High performance (GE Healthcare). His-tag-binding protein was eluted with elution buffer [50 mM NaH_2_PO_4_, 300 mM NaCl, and 500 mM imidazole (pH 7.5)], dialyzed against PBS using a Mini Dialysis kit (GE Healthcare), and the protein was stocked at -80°C until use.

### Enzyme-linked immunosorbent assay (ELISA)

Serum samples were assayed by ELISA using plates coated with purified RRP8 or TNP1 proteins as described previously [50]. Briefly, flat-bottom 96-well plates (Thermo Scientific Japan, Yokohama, Japan) were coated with the recombinant proteins purified by wheat germ cell-free synthesis at 500 ng per well diluted in 0.05 M carbonate buffer (pH 9.6) at 4°C overnight. The remaining free binding sites were blocked with blocking reagents (Blocking One; Nacalai Tesque) for 1 h at room temperature. Wells were incubated with serum samples diluted 1:500 for 2 h and subsequently with HRP-conjugated goat anti-human IgG (Fab)_2_ (Abcam, Cambridge, UK) for 1 h. The antibody binding was visualized by incubation with tetramethylbenzidine (SurModics, Eden Prairie, MN, USA). The reaction was stopped with stop solution (SurModics). The optical density at 450 nm (OD450) was then read with a FlexStation 3 (Molecular Devices Japan, Tokyo, Japan). All incubations were followed by three washes with PBST. Samples were tested in duplicate, and the antibody units were calculated from the OD450 results using a standard curve obtained from serial concentrations of serum from each LN patient containing a high titer of each antibody.

The anti-dsDNA antibodies in sera from humans and mice were analyzed using commercial ELISA kits (MBL, Nagoya, Japan) in accordance with the manufacturer’s protocol. Anti-RRP8 and anti-TNP1 antibodies in sera from mice were measured as described above, using HRP-conjugated goat anti-mouse IgG (Fab)_2_ (GeneTex, Irvine, CA, USA). We estimated the glomerular filtration rate (GFR) using the following equation: eGFR (mL/min/1.73 m2) = 194 x serum creatinine (-1.094) x age (-0.287) x 0.739 (if female) [51].

### Induction of glomerulonephritis in mice

Female 8-week-old C57BL/6 mice were purchased from Clea Japan (Tokyo, Japan), and housed under specific pathogen-free conditions. The mice were immunized subcutaneously with 20 μg of purified protein (RRP8 or TNP1) in 100 μL Freund’s complete adjuvant on day 0 and in Freund’s incomplete adjuvant on day 14, and then boostered intravenously from the tail vein every 10 days. Proteinuria was measured semiquantitatively using urine dipsticks (Siemens Healthcare Diagnostics Japan, Tokyo, Japan). After detection of proteinuria (7–9 injections), the mice were sacrified and their sera and the organs such as kidneys, lungs, spleens and livers were stocked.

### Immunofluorescence staining

The rabbit anti-human RRP8 (Atlas Antibodies, Stokholm, Sweden) and rabbit anti-human TNP1 (Atlas Antibodies) antibodies were labeled with a Zenon Alexa Fluor 546 rabbit IgG Labeling Kit (Life Technologies Japan, Tokyo, Japan) in accordance with the manufacturer’s instructions. Formalin-fixed, paraffin-embedded kidney sections of biopsy or autopsy specimens from LN patients were processed for double immunofluorescence labeling. Briefly, deparaffinized sections were incubated with 10% normal donkey serum for 15 min at room temperature, followed by incubation with rabbit anti-human IgG (DAKO Japan, Kyoto, Japan) or rabbit anti-human C3 antibody (DAKO Japan) for 1 h at room temperature. After washing with Tris-buffer saline and 0.05% (v/v) Tween 20, the specimens were incubated with FITC-conjugated donkey anti-rabbit IgG antibody for 1 h at room temparature. Subsequently, the specimens were incubated with Zenon Alexa Fluor 546-labeled anti-RRP8 or anti-TNP1 antibody at 4°C overnight, then observed and photographed with a BZ-9000 fluorescence microscope (Keyence Japan, Osaka, Japan).

Cryostat tissue sections from mice were also analyzed by double immunofluorescence using a combination of anti-human RRP8 or anti-human TNP1 antibody and anti-mouse IgG antibody. The rabbit anti-human RRP8 and rabbit anti-human TNP1 antibodies were labeled with Zenon Alexa Fluor 568 rabbit IgG Labeling kit (Life technologies Japan), and the rabbit anti-mouse IgG antibody (Bethyl Laboratories, Montgomery, TX, USA) was labeled with Zenon Alexa Fluor 488 rabbit IgG Labeling kit. The sections were blocked with 2% (w/v) BSA in PBS for 1 h at room temperature and then permeabilized for 1 h at room temperature using 4% (w/v) BSA and 0.2% (v/v) Triton X-100 in PBS. Subsequently, the sections were incubated with Zenon Alexa Fluor 568-labeled anti-human RRP8 or anti-human TNP1 antibody and Zenon Alexa Fluor 488-labeled anti-mouse IgG antibody for 2 h at room temperature.

The phenotypes of the infiltrating cells in renal tissues were also analyzed by immunofluorescence staining using cryostat kidney sections and the following Alexa Fluor 488-labeled antibodies (BioLegend Japan KK, Tokyo, Japan): hamster anti-mouse CD3ε monoclonal antibody (MoAb) (145-2C11); rat anti-mouse CD4 MoAb (GK1.5); rat anti-mouse CD8a MoAb (53–6.7); rat anti-mouse CD11b MoAb (M1/70); and rat anti-mouse Ly-6G/Ly-6C (Gr-1) MoAb (RB6-8C5). Quantitative analysis of infiltrating cells in glomerular and tubulointerstitial areas was performed as follows: glomerular areas, cell counts in 20 random glomeruli; and tubulointerstitial areas, 10 random fields at x400 magnification.

### Real-time PCR for cytokines in the mouse kidney

The expressions of cytokines were quantified by real-time PCR (qRT-PCR). Briefly, for qRT-PCR analysis, total RNAs were extracted from snap-frozen kidneys of all mice in each group using Sepasol-RNA I Super G reagent (Nacalai Tesque). The cDNA was prepared with a SuperScript III CellsDirect cDNA synthesis system (Invitrogen, Carlsbad, CA, USA). The expressions of the cytokine genes were quantified using a QuantiTect SYBR Green PCR kit (Qiagen, Valencia, CA, USA) in a 7500 Real Time PCR System (Applied Biosystems, Tokyo, Japan), and corrected with a hypoxanthine phosphoribosyl transferase 1 (HPRT1) control. Amplifications were done in a total volume of 25 μL for 45 cycles of 15 s at 95°C and 1 min at 60°C. Samples were run in triplicate and their relative expression was determined by normalizing the expression of each target to HPRT1 and then comparing this normalized value with the normalized expression in a reference control sample to calculate the fold change value. The primers for the amplicons spanned intron/exon boundaries to minimize any amplification of genomic DNA. The primer sequences were as follows: IL-4, forward 5’-ACGGAGATGGATGTGCCAAAC-3’ and reverse 5’-AGCACCTTGGAAGCCCTACAGA-3’; IL-6, forward 5’-CAACGATGATGCACTTGCAGA-3’ and reverse 5’-CTCCAGGTAGCTATGGTACTCCAGA-3’; IL-17A [52], forward 5’- GACCAGGATCTCTTGCTGGA-3’ and reverse 5’- GGACTCTCCACCGCAATGA-3’; IFN-γ, forward 5’-CGGCACAGTCATTGAAAGCCTA-3’ and reverse 5’-GTTGCTGATGGCCTGATTGTC-3’; TNF-α, forward 5’-AGCGAGGACAGCAAGGGACTA-3’ and reverse 5’-GAGCTATTTCCAAGATGTTCTGGAG-3’; and HPRT1, forward 5’-TTGTTGTTGGATATGCCCTTGACTA-3’ and reverse 5’-AGGCAGATGGCCACAGGACTA-3’.

## Immunoelectron microscopy

The mouse kidneys were fixed for 30 min at 4°C in 4% paraformaldehyde and 0.1% glutaraldehyde in phosphate buffer (0.1 M sodium/potassium phosphate buffer, pH 7.3), dehydrated, and embedded in LR White resin (Polysciences, Warrington, PA. USA). Ultrathin sections (<60~80 nm), cut with a Leica Ultracut S (Leica, Wetzlar, Germany), were mounted on nickel grids. Nonspecific labeling was blocked by preincubation with 5% normal goat serum, 5% BSA, and 0.1% Tween 20 in PBS for 3 h at room temperature. For double immunogold labeling, the sections were first incubated with rabbit anti-human RRP8 or rabbit anti-human TNP1 antibody (Atlas Antibodies) at 4°C overnight and washed three times in PBT (0.005% Tween 20 in PBS). Then the sections were incubated with 10-nm-gold-conjugated anti-rabbit IgG antibody and 5-nm-gold-conjugated anti-mouse IgG antibody (British BioCell Int., Dundee, UK), washed again twice in PBT, then twice in distilled water, and air-dried. Finally, the sections were contrasted with a saturated solution of uranyl acetate in water (20 min) and observed in a JEM1230 electron microscope (JEOL, Tokyo, Japan) equipped with a CCD camera (Model SC1000; Gatan, Pleasanton, CA, USA).

### PCR detection of RRP8 and TNP1 in the human tissues

To examine the expressions of RRP8 and TNP1 in various human tissues, we performed PCR using MTC (multiple tissue cDNA) cDNA panels (Clontech, Mountain View, CA, USA) in accordance with the manufacturer’s instructions. The primer sequences were as follows: RRP8, forward 5’- GACCCTCATGTTCGAAGAGC-3’ and reverse 5’-AACCAGGTCTTGGCTCACAG-3’; TNP1, forward 5’-TGGCAGAACTTACCATGTCG-3’ and reverse 5’-GGGGAAAAACAGCCAACATA-3’; and glyceraldehyde-3-phosphate dehydrogenase (G3PDH), forward 5’-TGAAGGTCGGAGTCAACGGATTTGGT-3’ and reverse 5’-CATGTGGGCCATGAGGTCCACCAC-3’. The required fragments were amplified using TaKaRa Ex Taq (Takara Bio, Shiga, Japan). cDNA was subjected to 35 cycles of PCR (94°C for 30 s, and 68°C for 2 min) to detect TNP1 and G3PDH, and to 35 cycles (94°C for 30 s, and 68°C for 4 min) to detect RRP8.

### Statistical analysis

Comparisons between groups were examined using the Mann-Whitney U test. Correlations were expressed as Spearman rank correlation coefficients. Differences at probability (p) values of <0.05 were considered significant.

## Results

### Screening of autoantigens associated with LN using a biotinylated human autoantigen library

To screen LN-associated autoantigens, we selected serum samples from two SLE patients: patient A with nephritis but without serositis, and patient B with serositis but without nephritis (S2 Table). Using the AlphaScreen assay, a total of 2,296 biotinylated proteins were evaluated for their reactivity with antibodies in the serum from both patients in the active and inactive disease phases. Consequently, in comparison with sera taken in the inactive phase, 456 and 525 proteins showed higher positive signals in the active phase in patients A and B, respectively. Among these proteins, we selected 17 that showed a high luminescence signal (more than 3) in patient A and higher positive signals in the serum from patient A than in the serum from patient B (Table 1).

**Table 1:** Screening of 17 autoantigens associated with LN using a biotinylated human autoantigen library.

Gene	Protein name	Luminescence signal		Molecular mass (kDa)	Expected isoelectric point
		patient A	patient B		
**RRP8**	Ribosomal RNA-processing protein 8	37.94	7.88	50.7	9.87
**GABARAPL2**	Gamma-aminobutyric acid receptor-associated protein-like 2	25.93	2.23	13.7	8.22
**PRM2**	Protamine-2	17.44	1.27	13.1	12.08
**TNP1**	Spermatid nuclear transition protein 1	15.17	1.17	6.4	12.33
**USP10**	Ubiquitin carboxyl-terminal hydrolase 10	11.97	8.10	87.1	5.06
**FKBP4**	Peptidyl-prolyl cis-trans isomerase FKBP4	10.71	2.71	51.8	5.22
**CLIC5**	Chloride intracellular channel protein 5	8.47	1.78	46.5	4.55
**UBL5**	Ubiquitin-like protein 5	8.15	1.16	8.5	8.50
**TAF11**	Transcription initiation factor TFIID subunit 11	7.78	5.22	23.3	4.61
**JSRP1**	Junctional sarcoplasmic reticulum protein 1	7.78	5.04	36.3	9.89
**SAMHD1**	Deoxynucleoside triphosphate triphosphohydrolase SAMHD1	7.02	3.44	72.2	6.86
**ADAP2**	Arf-GAP with dual PH domain-containing protein 2	5.15	1.42	44.3	9.71
**TRIP10**	Cdc42-interacting protein 4	5.05	1.37	68.4	5.49
**ETS2**	Protein C-ets-2	4.86	1.59	53.0	4.76
**PEX26**	Peroxisome assembly protein 26	4.53	1.68	33.9	6.09
**NHP2L1**	NHP2-like protein 1	4.19	2.58	14.2	8.41
**PRM1**	Sperm protamine P1	3.64	1.11	6.8	12.25

Luminescence signal was calculated as the signals/noise ratio. Expected isoelectric point was estimated using an online calculator (http://isoelectric.ovh.org/).

### Identification of LN-associated autoantigens by immunoprecipitation

**Fig 1:**
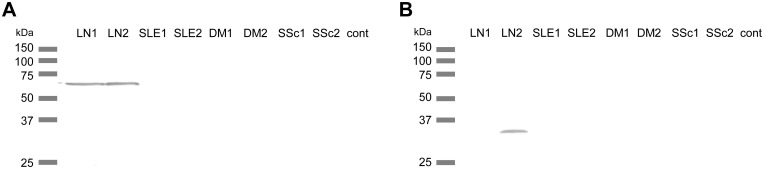
Immunoprecipitation of RRP8 and TNP1 with sera from patients with rheumatic diseases. Reprisentative photographs are shown. **(A)** RRP8 (50.7 kDa) **(B)** TNP1 (GST-tagged, 32.4 kDa). LN; active lupus nephritis, SLE; systemic lupus erythematosus without active lupus nephritis, DM; dermatomyositis, SSc; systemic sclerosis, cont; healthy control.

The biotinylated proteins of 17 autoantigens were prepared using the wheat germ cell-free protein synthesis system. Immunoprecipitation analysis using these biotinylated proteins was performed using sera from patients with the following diseases: 5 with active LN, 5 with SLE without nephritis, 5 with DM/PM, and 5 with SSc (S3 Table). Antibodies against RRP8, TNP1, sperm protamine P1 (PRM1), and protamine 2 (PRM2), were detected in the sera from SLE patients, while antibody against junctional sarcoplasmic reticulum protein 1 (JSRP1) was detected not only in the sera from SLE patients but also in sera from DM and SSc patients. The antibodies against PRM1 and PRM2 were detected in the sera from SLE patients with and without nephritis, although these positive signals were weak. Antibodies against RRP8 and TNP1 were detected specifically in patients with LN. As shown in Fig 1A and 1B, two proteins, RRP8 and TNP1, reacted with the sera from 2 and 1 LN patients, respectively. These two proteins did not react with any serum from healthy controls, SLE patients without nephritis, or DM/PM and SSc patients used in this experiment. These findings suggest that RRP8 and TNP1 are associated with LN.

### Deposition of anti-RRP8 and anti-TNP1 antibodies in glomeruli of LN patients

**Fig 2:**
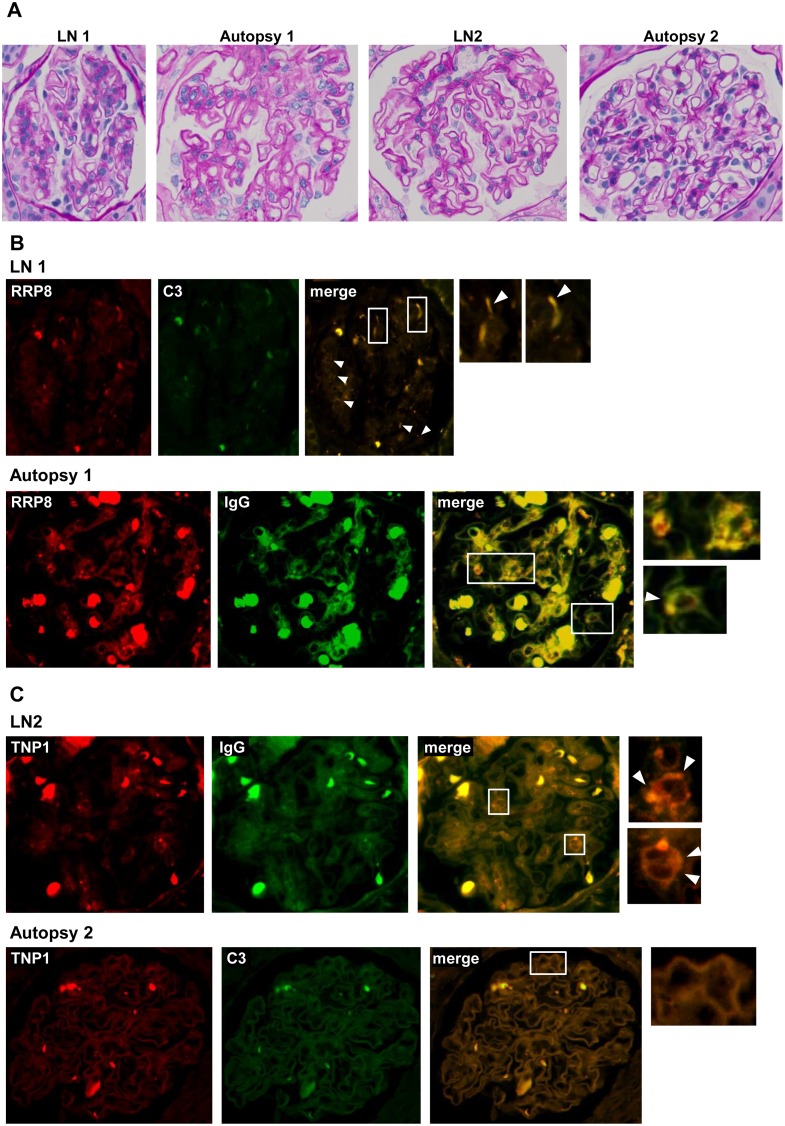
Double immunofluorescence of RRP8 or TNP1 and IgG or C3 in renal sections from patients with LN. Formalin-fixed, paraffin-embedded sections of biopsy or autopsy specimens from LN patients were processed for double immunofluorescence staining. Representative photographs are shown. The specimens were stained with Alexa 546-conjugated anti-RRP8 or anti-TNP1 antibodies (red) and with FITC-conjugated anti-IgG or anti-C3 antibodies (green). **(A)** The same glomeruli were stained with periodic acid-Schiff. **(B)** Co-presence of RRP8 and C3 was detected along the sub-epithelial area of the glomeruli showing a dotted pattern (arrowhead) in LN1. Also in Autopsy1, both RRP8 and IgG signals were detected in the mesangial area as well as in the sub-epithelial area (arrowhead). **(C)** Co-presence of TNP1 and IgG was revealed along the basement membrane showing a linear pattern in LN2, as was the case for TNP1 and C3 in Autopsy2. The bold and strong autofluorescence in each of the panels is mainly due to red blood cells.

To reveal whether anti-RRP8 and anti-TNP1 antibodies are deposited in glomeruli of LN patients, double immunofluorescence was performed using a combination of anti-RRP8 or anti-TNP1 antibodies and anti-IgG or anti-C3 antibodies. Kidney sections obtained by renal biopsy and autopsy from LN patients were analyzed in this study. Paraffin sections, and not frozen sections, were used as no frozen sections had been stocked. Therefore, nonspecific fluorescence due to red blood cells was evident (S1 Fig). As shown in Fig 2, in LN1 and Autopsy1, coexistence of RRP8 and IgG or C3 was detected in the sub-epithelial area of the glomeruli, showing a dotted pattern, and in the mesangial and sub-epithelial areas, respectively. On the other hand, in LN2 and Autopsy2, coexistence of TNP1 and IgG or C3 was revealed along the basement membrane, exhibiting a linear pattern. Anti-RRP8 antibody was deposited in glomeruli of 3 LN patients, while anti-TNP1 antibody was deposited in glomeruli of 3 LN patients (Table 2). These findings confirmed that anti-RRP8 and anti-TNP1 autoantibodies were deposited as IC in the glomeruli of some LN patients.

**Table 2:** Deposition of RRP8 and/or TNP1 in renal sections obtained by biopsy and autopsy from LN patients.

Case No.		RRP8	TNP1
LN1	Class III(A)+V	++	
LN2	Class III(A)	+	++
LN3	Class IV-G(A)+V		+
LN4	Class II		
LN5	Class III(A)+V		
LN6	Class III(A/C)		
LN7*	Class V	not examined	
Autopsy1	Class II	+	
Autopsy2	Class III(A/C)+V		+
Autopsy3	Class III(C)		

*Double immunofluorescence in kidney section of LN7 was not performed because of poor sample.

### Serum levels of anti-RRP8 and anti-TNP1 antibodies in patients with rheumatic diseases

**Fig 3:**
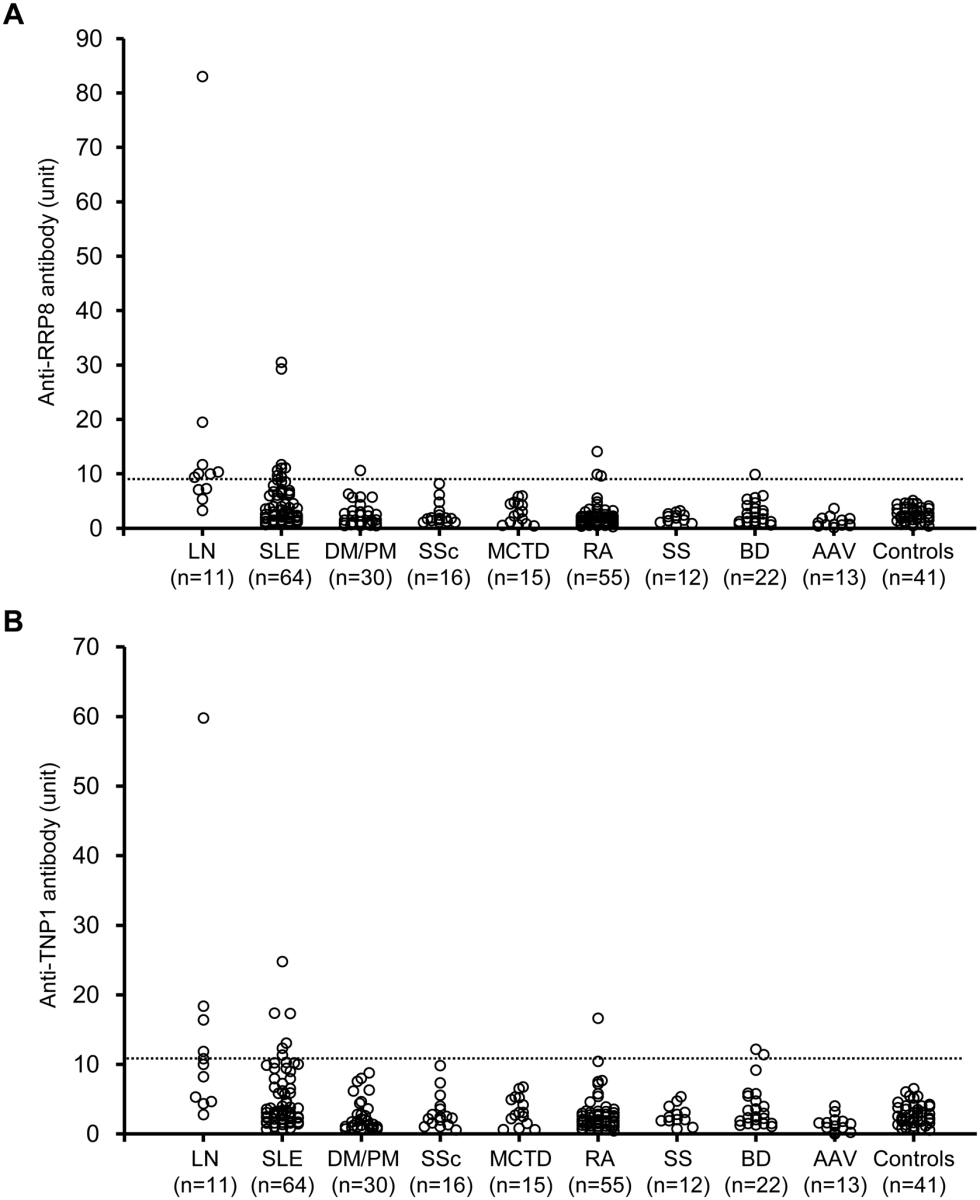
Serum levels of anti-RRP8 (A) and anti-TNP1 (B) antibodies in patients with rheumatic diseases. Cut-off values for positivity (9.1 units for anti-RRP8 antibody and 10.6 units for anti-TNP1 antibody) are indicated by the dotted lines. LN, lupus nephrits; SLE, systemic lupus erythematosus without LN; DM, dermatomyositis; PM, polymyositis; SSc, systemic sclerosis; MCTD, mixed connective tissue disease; RA, rheumatoid arthritis; SS, Sjögren syndrome; BD, Behçet’s disease; AAV, anti-neutrophil cytoplasmic antibody-associated vasculitis.

We compared the circulating levels of anti-RRP8 and anti-TNP1 antibodies among patients with rheumatic diseases using ELISA. We analyzed a total of 238 serum samples obtained from patients with various rheumatic diseases (S4 Table). When the cut-off value was considered to be the mean plus 5 standard deviations (SD) for 41 healthy control sera, the cut-off level for anti-RRP8 and anti-TNP1 antibodies was set at 9.1 units and 10.6 units, respectively. Among these serum samples, anti-RRP8 antibody was positive in 20 patients; 15 SLE, 1 DM/PM, 3 RA and 1 BD (Fig 3A). Among the SLE patients, the positive rate of anti-RRP8 antibody was significantly higher in LN than that in SLE without nephritis (63.6% (n = 7) vs 12.5% (n = 8), respectively; p = 0.00017, odds ratio 12.25, 95% confidence interval 2.9–51.4). On the other hand, 14 were positive for anti-TNP1 antibody; 11 SLE, 1 RA and 2 BD (Fig 3B). The frequency of anti-TNP1 antibody was significantly higher in the SLE patients with nephritis than in those without nephritis (45.5% (n = 5) vs 9.4% (n = 6), respectively; p = 0.002, odds ratio 8.06, 95% confidence interval 1.8–34.5). These findings indicate that both anti-RRP8 and anti-TNP1 antibodies are strongly associated with LN.

**Fig 4:**
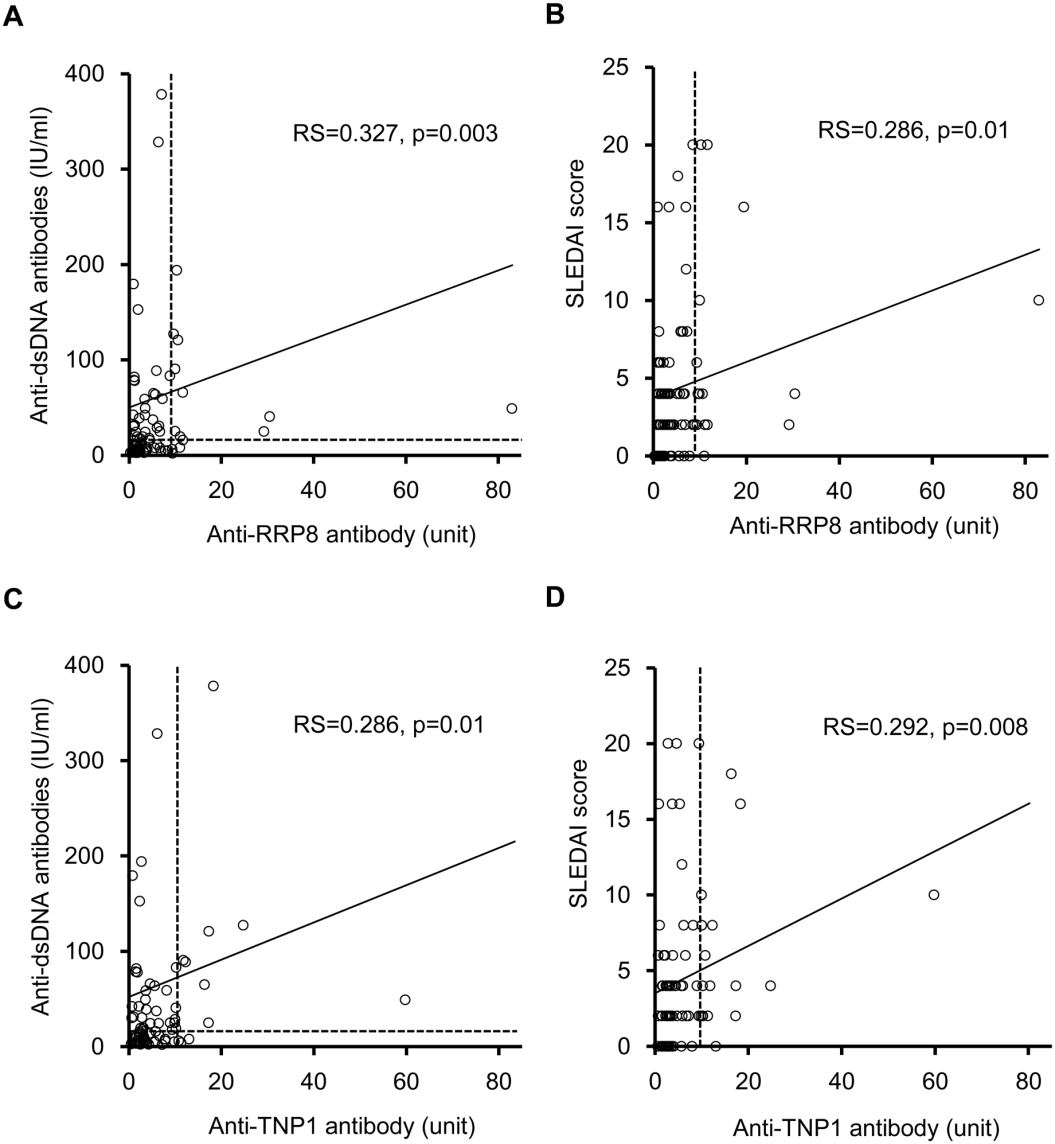
Relationship between anti-RRP8 or anti-TNP1 antibody and clinical findings in SLE patients. **(A)** anti-RRP8 antibody and anti-dsDNA antibodies, **(B)** Anti-RRP8 antibody and SLEDAI, **(C)** anti-TNP1 antibody and anti-dsDNA antibodies, **(D)** anti-TNP1 antibody and SLEDAI. SLEDAI, systemic lupus erythematosus disease activity index. Correlations were expressed as Spearman rank correlation coefficients. P values of <0.05 were considered significant.

In the SLE cohort, the levels of anti-dsDNA antibodies as well as the SLEDAI scores were weakly correlated with the levels of anti-RRP8 and anti-TNP1 antibodies (Fig 4, Table 3). Complement factor 3 (C3) was correlated with anti-TNP1 antibody, and complement factor 4 (C4) was correlated with both anti-RRP8 and anti-TNP1 antibodies. No correlation with eGFR was evident (Table 3).

**Table 3:** Correlation between anti-RRP8 or anti-TNP1 antibody level and clinical findings in SLE patients.

	Anti-RRP8 antibody		Anti-TNP1 antibody	
	Spearman’s rs	p value	Spearman’s rs	p value
SLEDAI	0.286	0.0096	0.292	0.0082
anti-dsDNA antibody (IU/ml)	0.327	0.0031	0.286	0.0096
C3 (mg/dL)	-0.187	0.0902	-0.228	0.0388
C4 (mg/dL)	-0.363	0.0010	-0.331	0.0027
eGFR (mL/min/1.73 m^2^)	0.208	0.0602	0.090	0.4131

Correlations were expressed as Spearman rank correlation coefficients. P values of <0.05 were considered significant.

**Fig 5:**
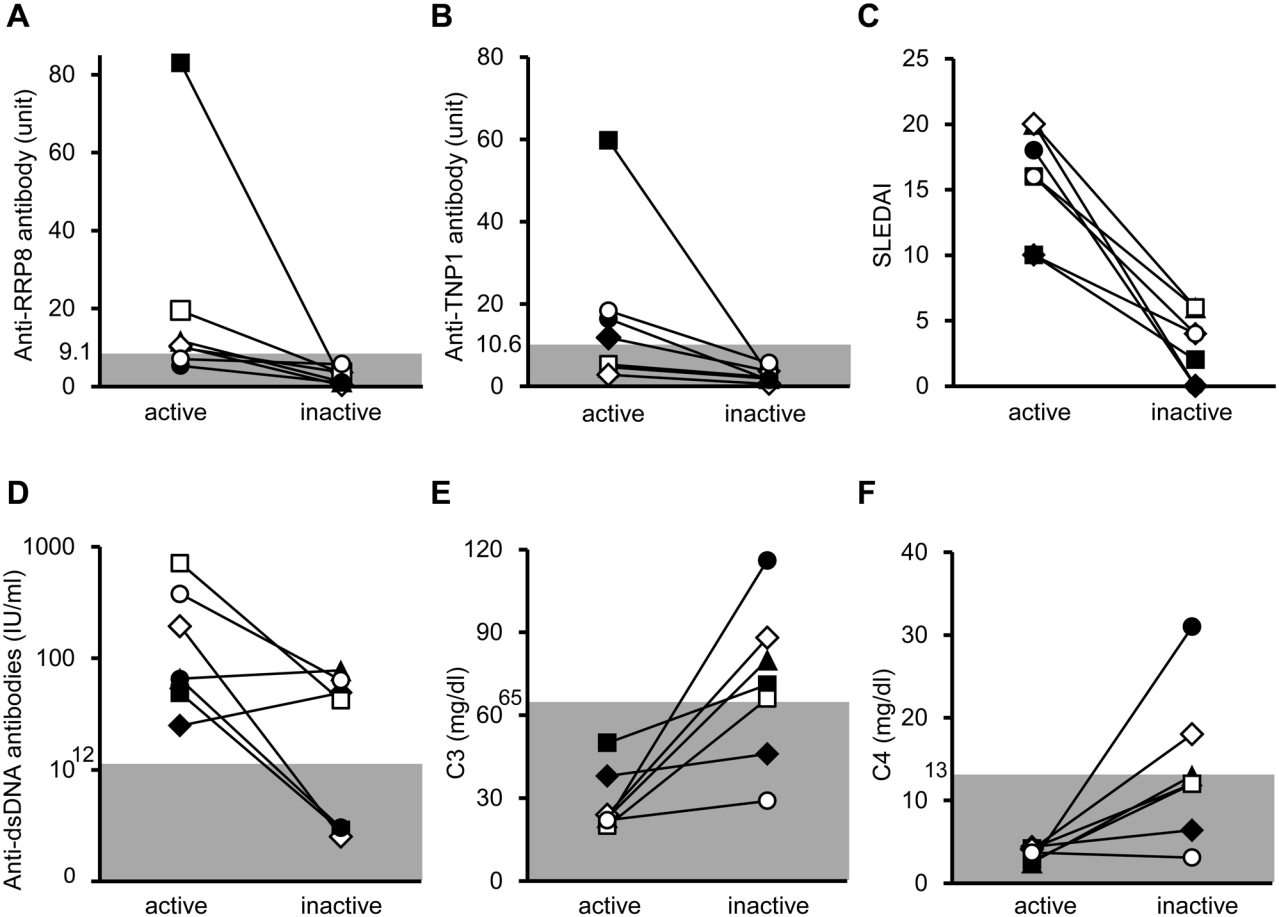
Changes in antibody levels and clinical findings in patients with lupus nephritis. ▲ LN1, ♦ LN2, ● LN3, ◊ LN4, ○ LN5, □ LN6, ■ LN7. **(A)** Anti-RRP8 antibody, **(B)** anti-TNP1 antibody, **(C)** SLEDAI, **(D)** anti-dsDNA antibody, **(E)** C3, and **(F)** C4. The active and inactive phases of LN were defined as 4≤ and 0 of the renal SLEDAI score, respectively.

Among 11 patients with active LN, 7 were compared for their levels of anti-RRP8 and anti-TNP1 antibodies between the active (at onset) and inactive (after remission) phases. In the other 4 patients, the sera after remission had not been stocked. Both the anti-RRP8 and anti-TNP1 antibodies were significantly decreased in the inactive phase (Fig 5). The SLEDAI scores and the levels of anti-dsDNA antibodies were also decreased, and the levels of C3 and C4 were increased significantly, although some patients still retained high titers of anti-dsDNA antibodies or hypocomplementemia even after improvement of nephritis. In LN1, the level of anti-RRP8 antibody decreased from 11.7 units to 1.2 units after remission, whereas that of anti-dsDNA antibodies increased from 65.7 IU/ml to 77.8 IU/ml. In LN2, the level of anti-TNP1 antibody decreased from 11.9 units to 3.7 units after remission, whereas the level of anti-dsDNA antibodies increased from 24.9 IU/ml to 49.1 IU/ml. These findings suggest that the levels of anti-RRP8 and anti-TNP1 antibodies do not correlate with those of anti-dsDNA antibodies in some LN patients.

### Induction of IC-deposited glomerulonephritis in mice by immunization with RRP8 or TNP1 antigen

In our first experiment, we examined whether anti-RRP8 and anti-TNP1 antibodies were present in the sera of MRL/lpr mice. MRL/lpr mice spontaneously develop glomerular disease, which becomes noticeable by the age of 12 weeks, in association with an increase of circulating ICs, autoantibody production and cytokine abnormalities, closely resembling those seen in human LN [53]. Consequently, anti-RRP8 and anti-TNP1 antibodies were not detectable in the sera of 8-week-old MRL/lpr mice. MRL/lpr mice showed a significant increase in both anti-RRP8 and anti-TNP1 autoantibodies at the ages of 12 weeks and 20 weeks (S2 Fig). However, we were unable to examine immune deposits of RRP8 and TNP1 in the kidneys of MRL/lpr mice, since no antibodies against mouse RRP8 or mouse TNP1 suitable for histological analysis were available commercially.

**Fig 6:**
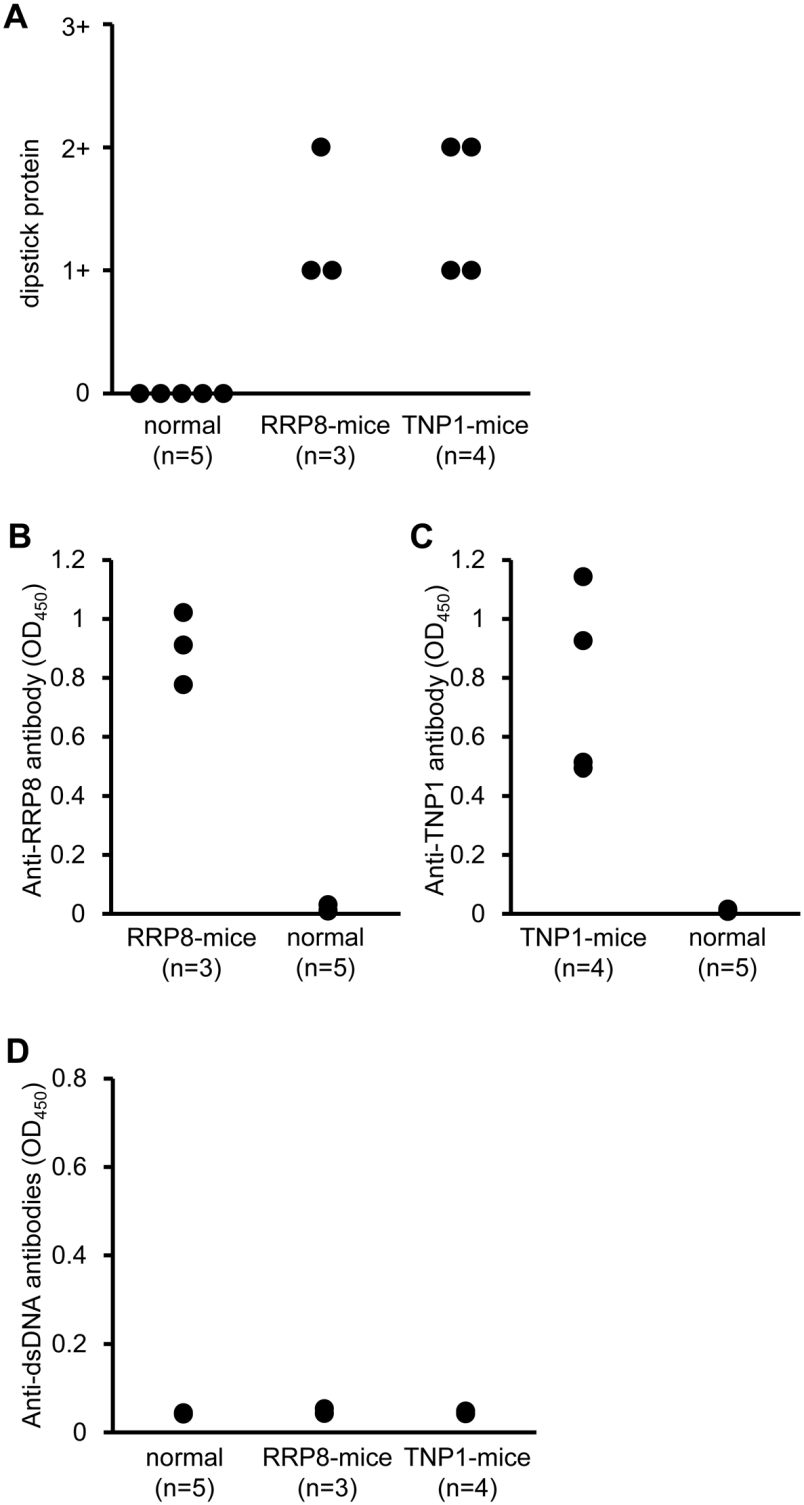
Measurement of proteinuria (A) and anti-RRP8 (B), anti-TNP1 (C) or anti-dsDNA (D) antibodies in murine serum samples. Proteinuria was assessed before sacrifice. The urine diluted 1:10 was examined and graded with a score of 0 (<30mg/dL); 1 (30-99mg/dL); 2 (100-299mg/dL); or 3 (300-999mg/dL). Serum samples were assayed by ELISA using purified human RRP8 or TNP1 protein. RRP8- or TNP1-mice; C57BL/6 mice injected repeatedly with RRP8 or TNP1, respectively. normal; normal C57BL/6 mice.

**Fig 7:**
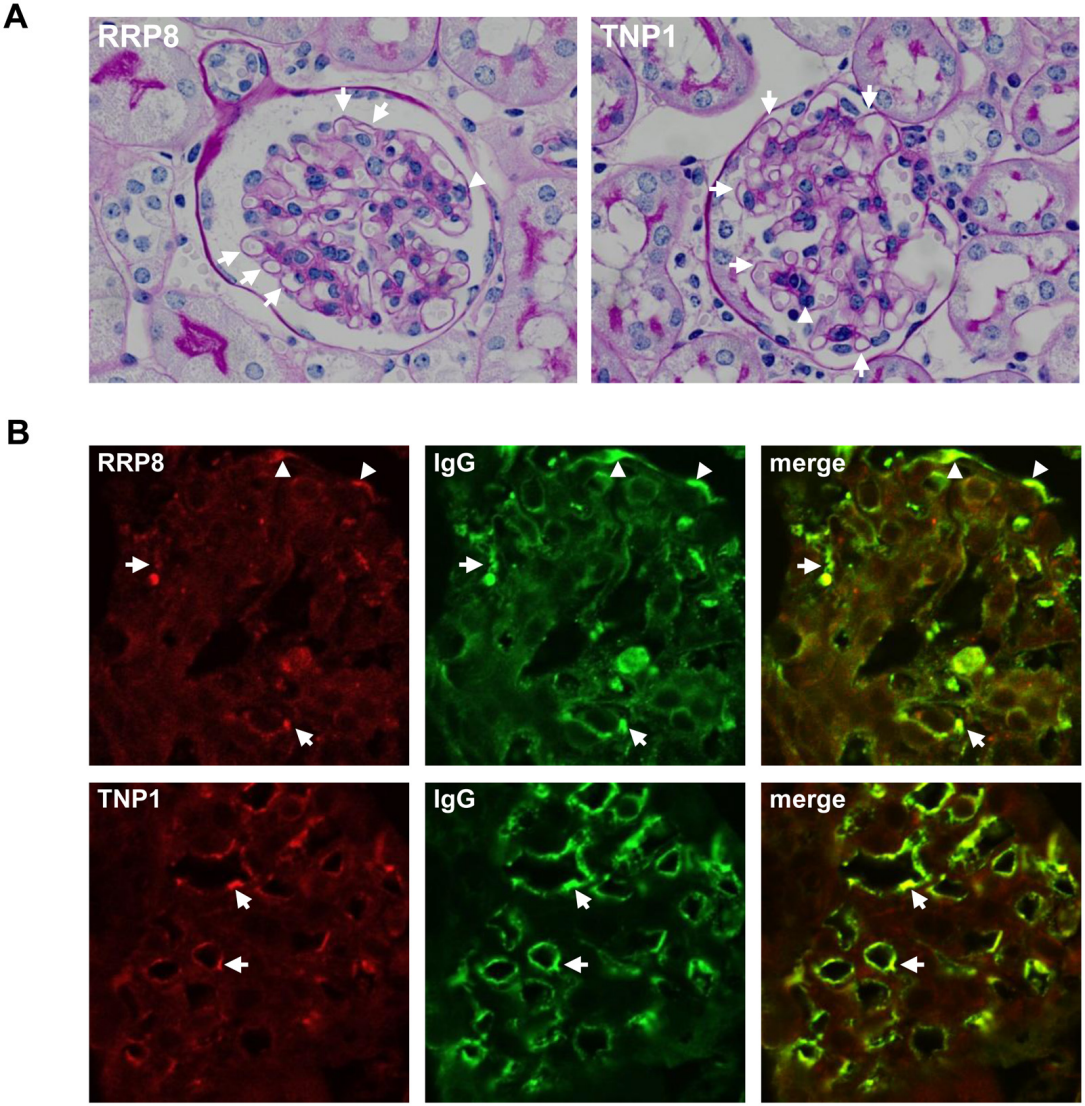
Double immunofluorescence of RRP8 or TNP1 and IgG in renal sections from RRP8-injected or TNP1-injected mice. Cryostat kidney sections from mice were analyzed by double immunofluorescence staining with a combination of anti-human RRP8 or anti-human TNP1 antibody and anti-mouse IgG antibody. The specimens were stained with Alexa 568-conjugated anti-RRP8 or anti-TNP1 antibodies (red) and with Alexa 488-conjugated anti-murine IgG antibody (green). **(A)** Photomicrograph of representative glomeruli of RRP8- and TNP1-injected mice (periodic acid-Schiff staining). Both manifested the slight enlargement of glomeruli with thickening of capillary wall (arrows) as well as the endocapillary proliferation with leukocytes (arrow head). **(B)** RRP8 signals coexisted with IgG signals in the sub-endothelial (arrows) and sub-epithelial (arrowhead) regions, exhibiting a granular pattern. On the other hand, TNP1 signals coexisted with IgG signals in the sub-epithelial region (arrows).

Next, we examined whether IC would be formed by injection of human RRP8 or TNP1 into C57BL/6 mice and whether glomerulonephritis would occur predominantly when IC was formed with RRP8 or TNP1. C57BL/6 mice were injected repeatedly with recombinant human RRP8 or TNP1 protein. C57BL/6 mice are not prone to autoimmune disease, but after 7–9 injections with RRP8 or TNP1, they demonstrated proteinuria (Fig 6A). In the sera of these injected mice, a high titer of anti-RRP8 or anti-TNP1 antibody was evident, but anti-dsDNA antibodies were negative (Fig 6B–6D). This indicates that RRP8 and TNP1 do not cross-react with anti-dsDNA antibodies. In kidneys, some glomeruli of RRP8-injected mice showed a slight enlargement of glomeruli with thickening of capillary wall and endocapillary proliferation with infiltrating leukocytes as same as those of TNP1-injected mice (Fig 7A). IC deposits in cryostat kidney sections were analyzed by double immunofluorescence using a combination of anti-RRP8 or anti-TNP1 antibody and anti-IgG antibody. As shown in Fig 7B, in RRP8-injected mice, co-presence of RRP8 and IgG was evident in sub-endothelial and sub-epithelial regions with a coarse granular pattern. In TNP1-injected mice, co-presence of TNP1 and IgG was also demonstrated as a granular pattern in the sub-endothelial regions of glomeruli.

**Fig 8:**
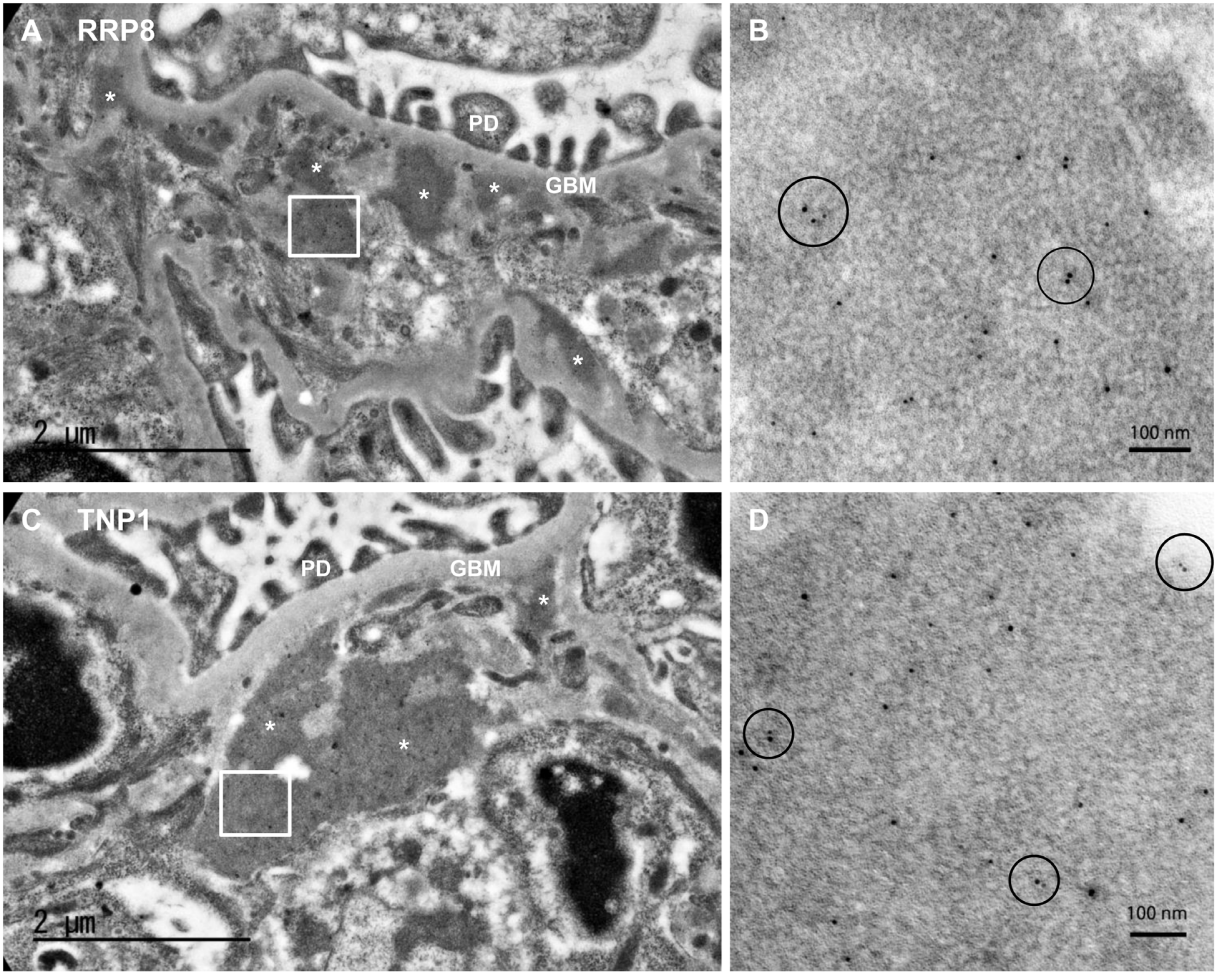
Immunoelectron microscopy of kidney sections from RRP8-injected (A and B) or TNP1-injected (C and D) mice. Photographs show the characteristic dense deposit in the subendothelial part especially at around the border of pericapillary and perimesangial areas. Panel B (anti-RRP8) and Panel D (anti-TNP1) show the high-power view of the deposition which manifests the adjacent (within 30nm) of RRP8 or TNP1 represented by 10-nm-gold particles and IgG represented by 5-nm-gold particles (circle area). *, electron-dense deposit; PD, podocyte; GBM, glomerular basement membrane.

Moreover, to determine the deposition of ICs involving RRP8 or TNP1, immunoelectron microscopy was performed. Both RRP8-injected and TNP1-injected mice revealed subendothelial electron-dense deposits in low-power view (Fig 8A and 8C), especially around the border of pericapillary and perimesangial areas. High magnification demonstrated adjacent RRP8 or TNP1 represented by deposition of 10-nm gold particles and IgG represented by 5-nm gold particles (Fig 8B and 8D). These findings also indicate the deposition of IC involving RRP8 or TNP1 in glomeruli. On the other hand, inflammatory changes such as vasculitis and infiltrating inflammatory cells were hardly observed in the lung, liver and spleen of both RRP8-injected and TNP1-injected mice (S3–S9 Figs). In addition, IC deposits with RRP8 or TNP1 were not detected in the lung, liver and spleen of both RRP8-injected and TNP1-injected mice by immunofluorescence staining (S3–S9 Figs). These findings indicate that the IC formed with RRP8 or TNP1 is deposited preferentially in glomeruli, rather than in other organs.

### Analysis of infiltrating cells and cytokine expression in the kidneys of RRP8-injected and TNP1-injected mice

**Fig 9:**
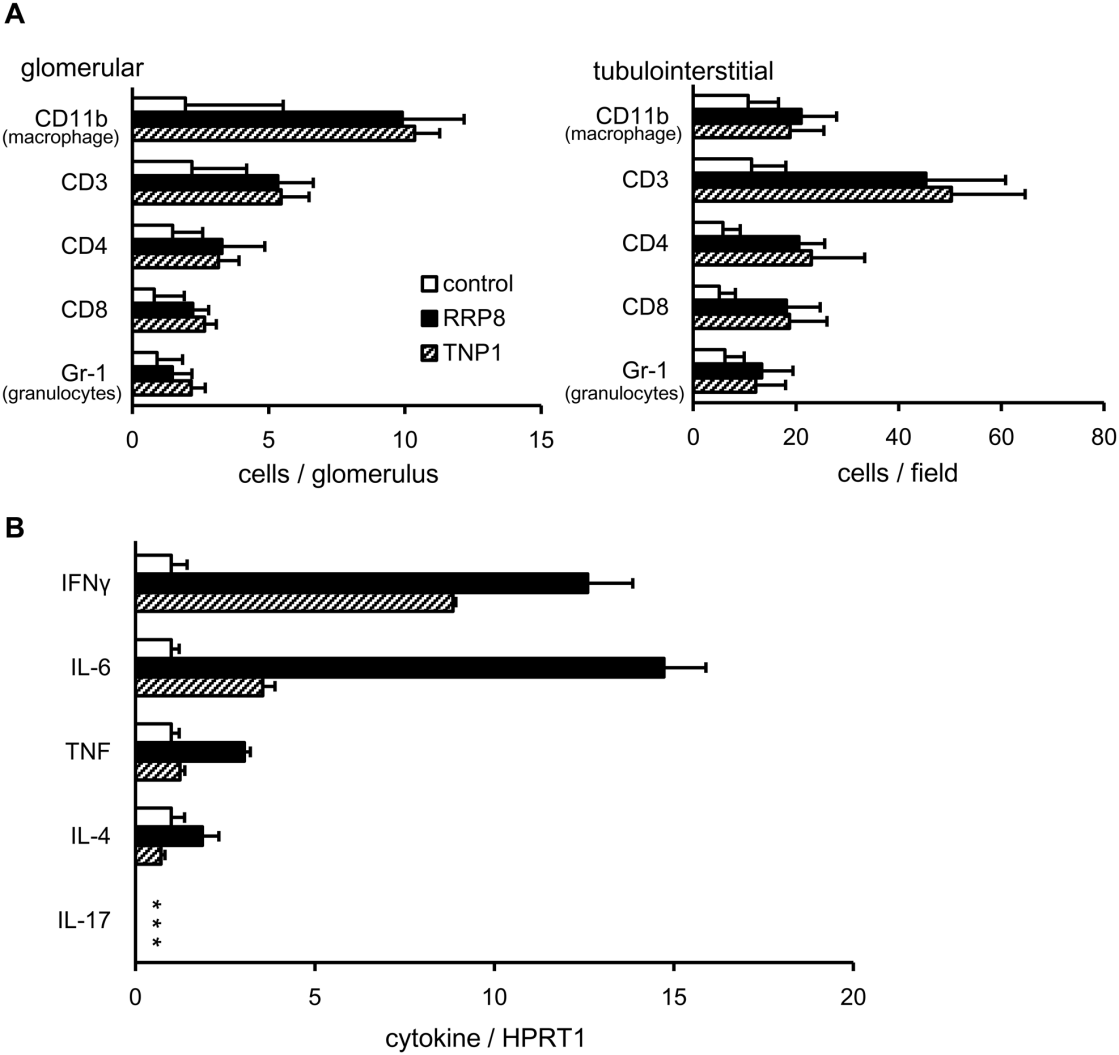
Analysis of infiltrating cells (A) and cytokine expression (B) in the kidneys of RRP8-injected and TNP1-injected mice. **(A)** Quantitative analysis of mononuclear cells in glomerular and tubulointerstitial areas was performed in control, RRP8-injected and TNP1-injected mice. Values are the mean and SD of infiltrating and parenchymal cells (glomerular, cell counts in 20 random glomeruli; and tubulointerstitial, 10 random fields at x400 magnification). **(B)** qRT-PCR analysis was performed on total RNA prepared from kidneys of all mice of each group. Results are calculated as a ratio of cytokine expression to the expression of HPRT1. * no positive signal with 45 cycles.

Next, we analyzed the infiltrating cells in glomerular and tubulointerstitial areas in control, RRP8-injected and TNP1-injected mice. As shown in Fig 9A, there was no significant difference in the composition of infiltrating cells in glomerular and tubulointerstitial areas between RRP8-injected and TNP1-injected mice. The majority of the infiltrating cells were CD11b-positive (macrophages) and CD3-positive (pan-T cells) in the kidneys of RRP8-injected and TNP1-injected mice. In the glomerulus, the numbers of infiltrating macrophages and T cells in RRP8-injected and TNP1-injected mice were 5-fold and 2-fold higher than those in control mice, respectively. In the tubulointerstitium, the numbers of infiltrating macrophages and T cells in RRP8-injected and TNP1-injected mice were 2-fold and 4-fold higher than those in control mice, respectively. These findings indicate that macrophages in the glomerulus and T cells in the tubulointerstitium predominantly infiltrate the kidneys of RRP8-injected and TNP1-injected mice. Among CD3-positive cells in the glomerulus and tubulointerstitium, the proportions of CD4- and CD8-positive cells were similar.

Using qRT-PCR, we compared the expression levels of cytokines in the kidneys among control, RRP8-injected and TNP1-injected mice. As shown in Fig 9B, the expression levels of IL-6 and IFN-γ in both RRP8-injected and TNP1-injected mice were increased significantly relative to those in control mice. Expression of IL-17A was undetectable with qRT-PCR using other two primer sets (data not shown). Furthermore, in RRP8-injected and TNP1-injected mice, the expression level of IFN-γ was high relative to those of IL-4 and IL-17A. This demonstrates that a Th1 immune response, rather than a Th2 or Th17 immune response, occurs predominantly in the kidneys of RRP8-injected and TNP1-injected mice.

## Discussion

In the present study, we identified two novel autoantigens—RRP8 and TNP1—associated with LN using an N-terminal biotinylated protein library created from a wheat cell-free protein production system. Our main findings were as follows: Circulating anti-RRP8 and anti-TNP1 autoantibodies were detected and deposited as ICs in the glomeruli of some LN patients; the positivity rates and levels of anti-RRP8 and anti-TNP1 autoantibodies were significantly higher in SLE patients with nephritis than in those without nephritis, and both antibodies were scarce or lacking in sera from patients with various other rheumatic diseases; ICs formed with human RRP8 or TNP1 after injection into C57BL/6 mice were deposited preferentially in glomeruli rather than in other organs; a Th1 immune response occurred predominantly in the kidneys of RRP8-injected and TNP1-injected mice; and these ICs may be trapped via anionic sites in the GBM, leading to deposition in glomeruli, since both antigens are cationic and their antibodies did not cross-react with dsDNA.

Renal injury in LN is initiated by deposition of autoantibodies and/or ICs in glomeruli, leading to activation of complement and macrophages, cell proliferation, production of extracellular matrix proteins, and pro-inflammatory cytokines and chemokines, which are linked through multiple mechanisms resulting in tubule damage, tubulointerstitial inflammation and fibrosis [4, 5]. Depending on the type, duration and severity of LN, immune deposits can be observed in the sub-epithelial, sub-endothelial, mesangial, and tubulointerstitial regions. Deposition of cationic immune deposits in the sub-endothelial or mesangial regions can initiate the recruitment of inflammatory cells and activation of resident endothelial and mesangial cells. Immune deposits in the sub-epithelial region are related to podocyte injury and proteinuria, whereas the glomerular basement membrane (GBM) acts as a barrier to leukocyte infiltration.

The main property of autoantibodies that is required for induction of nephritis is an ability to be deposited in the kidney and initiate inflammation. Anti-dsDNA antibodies are believed to play a central role in the pathogenesis of LN [4–6]. However, the exact mechanism through which anti-dsDNA antibodies are deposited in the kidney remains to be fully elucidated. Three mechanisms have been proposed to explain the propensity of anti-dsDNA antibodies to localize in the kidney. One is the formation of ICs with an ability to bind DNA/nucleosomes released from apoptotic cells [8, 54–56]. These ICs can deposit in the kidney and initiate an inflammatory cascade, interfering with the normal filtration barrier and causing proteinuria. Evidence for this theory is derived from the presence of DNA-anti-dsDNA complexes in the sera of LN patients, and elution of anti-dsDNA antibodies from diseased kidneys. The second theory, the planted antigen theory, suggests that anti-dsDNA antibodies form in the kidney by reacting with DNA/nucleosomes trapped in the GBM [57, 58]. The trapping of DNA/nucleosomes in the GBM has been attributed to charge interactions between DNA and the GBM. The third putative mechanism involves cross-reactivity between anti-dsDNA antibodies and kidney antigens including α-actinin, α-enolase, annexin II, annexin AI, and ribosomal P protein [21–28]. Although there is a general correlation between the presence of anti-dsDNA antibodies and SLE, several studies have demonstrated a discrepancy with LN. Alba et al. investigated different populations of autoantibodies in SLE patients with LN, as confirmed by renal biopsy [59]. Almost 99% of SLE patients with or without nephritis were positive for anti-nuclear antibodies. On the other hand, anti-dsDNA antibodies were positive in only 68% of patients with LN and about half of SLE patients without LN. This suggests that other autoantibodies contribute to the pathogenesis of LN.

Autoantibodies other than anti-dsDNA antibodies have been reported in LN patients [9–27]. Anti-C1q antibody is associated with LN [9–15]. C1q plays a crucial role in the clearance of apoptotic cells and immune complexes [60]. Both humans and mice with C1q deficiency have a risk of developing lupus-like syndromes and immune-mediated glomerulonephritis because of defective clearance of apoptotic cells, autoantigens, and immune complexes [61]. The prevalence of anti-C1q antibody in LN patients is 40–97%, and elevation of the anti-C1q titer predicts the development of LN or renal flares [15]. However, anti-C1q antibody has been reported in 45% of SLE patients without nephritis [13]. In addition, the presence of anti-C1q antibody has been demonstrated in patients with hypocomplementemic urticarial vasculitis, rheumatoid arthritis, non-SLE active glomerulonephritis, and human immunodeficiency virus infection [14]. Anti-nucleosome antibodies are also associated with LN, and their prevalence in LN patients is 60–90% [15–19]. Nucleosomes released by apoptotic cells are major T and B cell autoantigens in SLE [62]. A recent electron microscopy study of renal tissues from LN patients demonstrated that autoantibodies were colocalized with electron-dense extracellular deposits of chromatin, suggesting that intraglomerular membrane-associated nucleosomes are targeted by nephritogenic autoantibodies [63]. The frequency of anti-nucleosome antibodies in active SLE is similar to that of anti-dsDNA antibodies, and the titers of these autoantibodies have correlated with each other in most studies [16–19]. However, the performance of anti-nucleosome antibodies for predicting LN or renal flares does not appear to be superior to that of anti-dsDNA antibodies. Anti-Sm antibodies react with the common heptamer of U1, U2, U4, and U5 small-nuclear RNP, and anti-Sm antibodies are associated with the severity and activity of renal disease [8, 20]. Anti-dsDNA antibodies are also well known to cross-react with components of the cytoplasm and plasma membrane of the GBM, such as α-actinin, α-enolase, annexin II, annexin AI, and ribosomal P protein [21–27]. However, these autoantibodies do not have sufficient sensitivity and specificity for predicting LN or renal flares.

To obtain further clinical markers for diagnosis of LN and evaluation of its activity, we screened autoantigens reactive with serum antibodies from LN patients using a BPL-based screening method, which is useful for identification of autoantigen proteins reactive with autoantibodies recognizing conformational epitopes [29, 30]. After analysis using several methods, we identified two autoantigens, RRP8 and TNP1, that were associated with LN. Circulating anti-RRP8 and anti-TNP1 autoantibodies were recognized and deposited as ICs in the glomeruli of some LN patients. In addition, the positivity rates for both anti-RRP8 and anti-TNP1 autoantibodies were significantly higher in the LN group than in the SLE group without nephritis. From these findings, we concluded that RRP8 and TNP1 autoantigens were associated with LN. RRP8 is a cationic protein (pI = 9.87) consisting of 456 amino acids (a.a.) (molecular mass 50.7 kDa), localized mainly in the nucleolus [31]. The a.a. sequence of human RRP8 shows 76% homology with that of mouse RRP8, and expression of RRP8 was detected in all 16 tissues (S10 Fig). RRP8 is an essential component of the eNoSC complex and acts by recruiting histone-modifying enzymes [33–35]. The eNoSC complex is able to sense the energy status of cells: upon glucose starvation, elevation of the NAD (+)/NADP (+) ratio activates sirtuin 1, leading to histone H3 deacetylation followed by dimethylation of H3 at lysine 9 (H3K9me2) and the formation of silent chromatin in the rDNA locus. In the complex, RRP8 binds to H3K9me2 and acts as a methyltransferase. On the other hand, TNP1, also a cationic protein (pI = 12.33), consists of 55 a.a. (molecular mass 6.4 kDa) [32]. The a.a. sequence of human TNP1 shows 87% homology with its mouse counterpart. TNP1 is a basic protein expressed in the sperm nucleus and participates in chromatin condensation by replacing somatic-type histones with protamines during spermiogenesis [36]. TNP1 was highly expressed in testis, but moderately expressed in pancreas, thymus and prostate, and only weakly expressed in liver and ovary (S10 Fig) [64]. The GBM normally functions as both a size and a charge barrier to filtration of macromolecules. Especially, anionic sites located throughout all the layers of the GBM play a crucial role in the transglomerular passage of macromolecules, since it facilitates the passage of cationic macromolecules to a greater extent than that of anionic and neutral macromolecules [65, 66]. Both RRP8 and TNP1 are cationic proteins. Sera of mice immunized with recombinant RRP8 and TNP1 did not cross-react with dsDNA. These findings suggest that these proteins, when released from apoptotic cells, form ICs with each autoantibody and that these ICs may be trapped at anionic sites in the GBM, leading to deposition in glomeruli.

Both anti-RRP8 and anti-TNP1 autoantibodies were detected in the sera of MRL/lpr mice that developed nephritis, closely resembling the pattern seen in human LN. Therefore, we planned to investigate whether glomerulonephritis would be induced by injecting recombinant mouse RRP8 or mouse TNP1 protein into mice. However, we were unable to initiate this experiment because no antibodies against mouse RRP8 or mouse TNP1 suitable for histological analysis were available commercially. Therefore, we examined whether IC would be formed by injection of human RRP8 or TNP1 into C57BL/6 mice and whether glomerulonephritis would occur predominantly as a result of IC formation with RRP8 or TNP1. IC-deposited glomerulonephritis was induced in C56BL/6 mice by repeated stimulation with recombinant human RRP8 or TNP1 protein. Furthermore, IC formed with RRP8 or TNP1 was deposited preferentially in glomeruli rather than in other organs. In the kidneys of RRP8-injected and TNP1-injected mice, cells showing predominant infiltration were macrophages in glomeruli and T cells in the tubulointerstitium. The affected kidneys showed a predominant Th1 immune response, as the expression level of IFN-γ was high relative to that of IL-4 or IL-17A. Previous studies of infiltrating cells and immune balance in the kidneys of LN patients have indicated that in the glomeruli of patients with diffuse proliferative lupus nephritis (DPLN), the majority of infiltrating cells were macrophages, while T cells were somewhat rarer. In contrast, the major infiltrating cells in the interstitium were both T cells and macrophages, with a predominance of T cells, and a Th1 immune response was predominant in the renal tissues [67]. These findings resemble the features of glomerulonephritis seen in RRP8-injected and TNP1-injected mice.

In the present study, we identified two novel LN-associated autoantigens, RRP8 and TNP1. The levels of anti-RRP8 and anti-TNP1 autoantibodies did not correlate with those of anti-dsDNA antibodies in some LN patients. In addition, sera of mice immunized with recombinant RRP8 and TNP1 did not cross-react with dsDNA. Therefore, these autoantibodies may be useful for diagnosis and prediction of LN in subsets of SLE patients who are negative for anti-dsDNA antibodies. Because of the small number of LN patients investigated in this study, no definite conclusion can be drawn about the association between the levels of these autoantibodies and LN disease activity. Further investigations will be necessary to determine whether anti-RRP8 and anti-TNP1 antibodies can be used as biomarkers of LN disease activity, and for evaluating the performance of panels of LN-associated autoantibodies for diagnosis, monitoring, and prognostic stratification of patients with LN.

## Supporting Information

S1 Fig Nonspecific fluorescence of formalin-fixed, paraffin-embedded sections of kidneys. Formalin-fixed, paraffin-embedded sections of kidneys were stained with secondary antibody only; Alexa Fluor 546-conjugated anti-rabbit IgG antibody **(A)** or FITC-conjugated anti-rabbit IgG antibody **(B)**. Panel **(C)** is merged image. The strong autofluorescence in each panel is mainly due to red blood cells.
(TIF)

S2 Fig Measurement of anti-RRP8 (A) and anti-TNP1 (B) antibodies in the serum samples of MRL/lpr mice. The serum samples were prepared from MRL/lpr mice at ages 8, 12, and 20 weeks during the development of nephritis. Serum samples were assayed by ELISA using purified mouse RRP8 or TNP1 protein. The sera of C57BL/6 mice were used as a control.
(TIF)

S3 Fig Immunofluorescence (positive control) of RRP8 or TNP1 and IgG in the kidneys of RRP8-injected or TNP1-injected mice. (TIF)

S4 Fig Immunofluorescence of RRP8 and IgG in the lungs of RRP8-injected mice. Normal C57BL/6 mice were used as a control. Representative photographs are shown.
(TIF)

S5 Fig Immunofluorescence of TNP1 and IgG in the lungs of TNP1-injected mice. (TIF)

S6 Fig Immunofluorescence of RRP8 and IgG in the spleen of RRP8-injected mice. (TIF)

S7 Fig Immunofluorescence of TNP1 and IgG in the spleen of TNP1-injected mice. (TIF)

S8 Fig Immunofluorescence of RRP8 and IgG in the liver of RRP8-injected mice. (TIF)

S9 Fig Immunofluorescence of TNP1 and IgG in the liver of TNP1-injected mice. (TIF)

S10 Fig Expressions of TNP1 and RRP8 in the human tissues. The expressions of TNP1 and RRP8 were analyzed with PCR using MTC cDNA panels.
(TIF)

S1 Table Clinical and laboratory data of 11 LN patients. (PDF)

S2 Table Clinical and laboratory data of patient A and B. (PDF)

S3 Table Clinical and laboratory data of 20 patients analyzed with immunoprecipitation. (PDF)

S4 Table Information on 238 patients and 41 healthy individuals analyzed with ELISA. (PDF)

## References

[1] Rekvig OP, Van der Viag J. The pathogenesis and diagnosis of systemic lupus erythematosus: still not resolved. Semin Immunopathol. 2014;36: 301–311. 10.1007/s00281-014-0428-6 24763531

[2] Sherer Y, Gorstein A, Fritzler MJ, Shoenfeld Y. Autoantibody explosion in systemic lupus erythematosus: more than 100 different antibodies found in SLE patients. Semin Arthritis Rheum. 2004;34: 501–537. 15505768 10.1016/j.semarthrit.2004.07.002

[3] Waldman M, Madaio MP. Pathogenic autoantibodies in lupus nephritis. Lupus. 2005;14: 19–24. 15732283 10.1191/0961203305lu2054oa

[4] Schwartz N, Goilav B, Putterman C. The pathogenesis, diagnosis and treatment of lupus nephritis. Curr Opin Rheumatol. 2014;26: 502–509. 10.1097/BOR.0000000000000089 25014039 PMC4221732

[5] Lech M, Anders HJ. The pathogenesis of lupus nephritis. J Am Soc Nephrol. 2013;24: 1357–1366. 10.1681/ASN.2013010026 23929771 PMC3752952

[6] Deshmukh US, Bagavant H, Fu SM. Role of anti-DNA antibodies in the pathogenesis of lupus nephritis. Autoimmun Rev. 2006;5: 414–418. 16890896 10.1016/j.autrev.2005.10.010

[7] Du H, Chen M, Zhang Y, Zhao MH. Characterization of anti-mesangial cell antibodies and their target antigens in patients with lupus nephritis. J Clin Immunol. 2005;25: 279–285. 10.1007/s10875-005-4082-6 15981094

[8] Mannik M, Merrill CE, Stamps LD, Wener MH. Multiple autoantibodies form the glomerular immune deposits in patients with systemic lupus erythematosus. J Rheumatol. 2003;30: 1495–1504. 12858447

[9] Siegert CEH, Daha MR, Tseng MES, Coremans IEM, van Es LA, Breedveld FC. Predictive value of IgG autoantibodies against C1q for nephritis in systemic lupus erythematosus. Ann Rheum Dis. 1993;52: 851–856. 8311534 10.1136/ard.52.12.851 PMC1005214

[10] Sinico RA, Radice A, Ikehata M, Giammarresi G, Corace C, Arrigo G, et al Anti-C1q autoantibodies in lupus nephritis: prevalence and clinical significance. Ann N Y Acad Sci. 2005;1050: 193–200. 16014534 10.1196/annals.1313.020

[11] Trendelenburg M, Lopez-Trascasa M, Potlukova E, Moll S, Regenass S, Frémeaux-Bacchi V, et al High prevalence of anti-C1q antibodies in biopsy-proven active lupus nephritis. Nephrol Dial Transplant. 2006;21: 3115–3121. 16877491 10.1093/ndt/gfl436

[12] Moura CG, Lima I, Barbosa L, Athanazio D, Reis E, Reis M, et al Anti-C1q antibodies: association with nephritis and disease activity in systemic lupus erythematosus. J Clin Lab Anal 2009;23: 19–23. 10.1002/jcla.20280 19140207 PMC6648991

[13] Meyer OC, Nicaise-Roland P, Cadoudal N, Grootenboer-Mignot S, Palazzo E, Hayem G et al Anti-C1q antibodies antedate patent active glomerulonephritis in patients with systemic lupus erythematosus. Arthritis Res Ther. 2009;11: R87 10.1186/ar2725 19515233 PMC2714141

[14] Mok CC. Biomarkers for lupus nephritis: a critical appraisal. J Biomed Biotechnol. 2010;2010: 638413 10.1155/2010/638413 20414362 PMC2857808

[15] Yung S, Chan TM. Autoantibodies and resident renal cells in the pathogenesis of lupus nephritis: getting to know the unknown. Clin Dev Immunol. 2012;2012:139365 10.1155/2012/139365 22761629 PMC3386553

[16] Amoura Z, Koutouzov S, Chabre H, Cacoub P, Amoura I, Musset L, et al Presence of antinucleosome autoantibodies in a restricted set of connective tissue diseases: antinucleosome antibodies of the IgG3 subclass are markers of renal pathogenicity in systemic lupus erythematosus. Arthritis Rheum. 2000;43: 76–84. 10643702 10.1002/1529-0131(200001)43:1<76::AID-ANR10>3.0.CO;2-I

[17] Grootscholten C, Dieker JW, McGrath FD, Roos A, Derksen RH, van der Vlag J, et al A prospective study of anti-chromatin and anti-C1q autoantibodies in patients with proliferative lupus nephritis treated with cyclophosphamide pulses or azathioprine/methylprednisolone. Ann Rheum Dis. 2007;66: 693–696. 17135217 10.1136/ard.2006.065425 PMC1954637

[18] Bigler C, Lopez-Trascasa M, Potlukova E, Moll S, Danner D, Schaller M, et al Antinucleosome antibodies as a marker of active proliferative lupus nephritis. Am J Kidney Dis. 2008;51: 624–629. 10.1053/j.ajkd.2007.10.041 18371538

[19] Manson JJ, Ma A, Rogers P, Mason LJ, Berden JH, van der Vlag J, et al Relationship between anti-dsDNA, anti-nucleosome and anti-alpha-actinin antibodies and markers of renal disease in patients with lupus nephritis: a prospective longitudinal study. Arthritis Res Ther. 2009;11: R154 10.1186/ar2831 19828047 PMC2787270

[20] Korbet SM, Schwartz MM, Evans J, Lewis EJ. Severe lupus nephritis: racial differences in presentation and outcome. J Am Soc Nephrol. 2007;18: 244–54. 17167111 10.1681/ASN.2006090992

[21] Deocharan B, Qing X, Lichauco J, Putterman C. Alpha-actinin is a cross-reactive renal target for pathogenic anti-DNA antibodies. J Immunol. 2002;168: 3072–3078. 11884481 10.4049/jimmunol.168.6.3072

[22] Zhao Z, Weinstein E, Tuzova M, Davidson A, Mundel P, Marambio P, et al Cross-reactivity of human lupus anti-DNA antibodies with alpha-actinin and nephritogenic potential. Arthritis Rheum. 2005;52: 522–530. 15693007 10.1002/art.20862

[23] Renaudineau Y, Croquefer S, Jousse S, Renaudineau E, Devauchelle V, Guéguen P, et al Association of alpha-actinin-binding anti-double-stranded DNA antibodies with lupus nephritis. Arthritis Rheum. 2006;54: 2523–2532. 16868973 10.1002/art.22015

[24] Bruschi M, Sinico RA, Moroni G, Pratesi F, Migliorini P, Galetti M, et al Glomerular autoimmune multicomponents of human lupus nephritis in vivo: alpha-enolase and annexin AI. J Am Soc Nephrol. 2014;25: 2483–2498. 10.1681/ASN.2013090987 24790181 PMC4214525

[25] Yung S, Cheung KF, Zhang Q, Chan TM. Anti-dsDNA antibodies bind to mesangial annexin II in lupus nephritis. J Am Soc Nephrol. 2010;21: 1912–1927. 10.1681/ASN.2009080805 20847146 PMC3014006

[26] Sun KH, Liu WT, Tsai CY, Tang SJ, Han SH, Yu CL. Anti-dsDNA antibodies cross-react with ribosomal P proteins expressed on the surface of glomerular mesangial cells to exert a cytostatic effect. Immunology. 1995;85: 262–269. 7642215 PMC1383890

[27] Chindalore V, Neas B, Reichlin M. The association between anti-ribosomal P antibodies and active nephritis in systemic lupus erythematosus. Clin Immunol Immunopathol. 1998;87: 292–296. 9646839 10.1006/clin.1998.4541

[28] Yoshio T, Okamoto H, Onishi S, Minota S. Antiribosomal-P protein antibodies are associated with proliferative glomerulonephritis more strongly than with membranous glomerulonephritis in Japanese patients with systemic lupus erythematosus. Mod Rheumatol. 2012;22: 488–490. 10.1007/s10165-011-0549-x 22042098

[29] Matsuoka K, Komori H, Nose M, Endo Y, Sawasaki T. Simple screening method for autoantigen proteins using the N-terminal biotinylated protein library produced by wheat cell-free synthesis. J Proteome Res. 2010;9: 4264–4273. 10.1021/pr9010553 20575507 PMC2917173

[30] Mizutani Y, Matsuoka K, Takeda H, Shiogama K, Inada K, Hayakawa K, et al Novel approach to identifying autoantibodies in rheumatoid synovitis with a biotinylated human autoantigen library and the enzyme-labeled antigen method. J Immunol Methods. 2013;387: 57–70. 10.1016/j.jim.2012.09.011 23044167

[31] Ishikawa K, Nagase T, Nakajima D, Seki N, Ohira M, Miyajima N, et al Prediction of the coding sequences of unidentified human genes. VIII. 78 new cDNA clones from brain which code for large proteins in vitro. DNA Res. 1997;4: 307–313. 9455477 10.1093/dnares/4.5.307

[32] Luerssen H, Mattei MG, Schröter M, Grzeschik KH, Adham IM, Engel W. Nucleotide sequence of the gene for human transition protein 1 and its chromosomal localization on chromosome 2. Genomics. 1990;8: 324–330. 2249851 10.1016/0888-7543(90)90289-7

[33] Murayama A, Ohmori K, Fujimura A, Minami H, Yasuzawa-Tanaka K, Kuroda T, et al Epigenetic control of rDNA loci in response to intracellular energy status. Cell. 2008;133: 627–639. 10.1016/j.cell.2008.03.030 18485871

[34] Tsai YC, Greco TM, Boonmee A, Miteva Y, Cristea IM. Functional proteomics establishes the interaction of SIRT7 with chromatin remodeling complexes and expands its role in regulation of RNA polymerase I transcription. Mol Cell Proteomics. 2012;11: 60–76. 10.1074/mcp.A111.015156 22586326 PMC3418843

[35] Yang L, Song T, Chen L, Kabra N, Zheng H, Koomen J, et al Regulation of SirT1-nucleomethylin binding by rRNA coordinates ribosome biogenesis with nutrient availability. Mol Cell Biol. 2013;33: 3835–3848. 10.1128/MCB.00476-13 23897426 PMC3811878

[36] Meistrich ML, Mohapatra B, Shirley CR, Zhao M. Roles of transition nuclear proteins in spermiogenesis. Chromosoma. 2003;111: 483–488. 12743712 10.1007/s00412-002-0227-z

[37] Hochberg MC. Updating the American College of Rheumatology revised criteria for the classification of systemic lupus erythematosus. Arthritis Rheum. 1997;40: 1725 9324032 10.1002/art.1780400928

[38] Bohan A, Peter JB, Bowman RL, Pearson CM. Computer-assisted analysis of 153 patients with polymyositis and dermatomyositis. Medicine (Baltimore). 1977;56: 255–286. 327194 10.1097/00005792-197707000-00001

[39] Subcommittee for Scleroderma Criteria of the American Rheumatism Association Diagnostic and Therapeutic Criteria Committee. Preliminary criteria for the classification of systemic sclerosis (scleroderma). Arthritis Rheum. 1980;23: 581–590. 7378088 10.1002/art.1780230510

[40] Alarcon-Segovia D, Cardiel MH. Comparison between 3 diagnostic criteria for mixed connective tissue disease: study of 593 patients. J Rheumatol. 1989;16: 328–334. 2724251

[41] Aletaha D, Neogi T, Silman AJ, Funovits J, Felson DT, Bingham CO 3rd, et al 2010 Rheumatoid arthritis classification criteria: an American College of Rheumatology/European League Against Rheumatism collaborative initiative. Arthritis Rheum. 2010;62: 2582–2591. 10.1002/art.27580 20872595

[42] Vitali C, Bombardieri S, Jonsson R, Moutsopoulos HM, Alexander EL, Carsons SE et al Classification criteria for Sjögren's syndrome: a revised version of the European criteria proposed by the American-European Consensus Group. Ann Rheum Dis. 2002;61: 554–558. 12006334 10.1136/ard.61.6.554 PMC1754137

[43] International Study Group for Behçet’s Disease. Evaluation of diagnostic (“classification”) criteria in Behçet’s disease- towards internationally agreed criteria. Br J Rheumatol. 1992;31: 299–308. 1581771

[44] Exley AR, Bacon PA. Clinical disease activity in systemic vasculitis. Curr Opin Rheumatol. 1996;8: 12–18. 8867533 10.1097/00002281-199601000-00002

[45] Gladman DD, Ibañez D, Urowitz MB. Systemic lupus erythematosus disease activity index 2000. J Rheumatol. 2002;29: 288–291. 11838846

[46] Cornélis F, Fauré S, Martinez M, Prud'homme JF, Fritz P, Dib C, et al New susceptibility locus for rheumatoid arthritis suggested by a genome-wide linkage study. Proc Natl Acad Sci U S A. 1998;95: 10746–10750. 9724775 10.1073/pnas.95.18.10746 PMC27966

[47] Jawaheer D, Seldin MF, Amos CI, Chen WV, Shigeta R, Monterio J, et al A genomewide screen in multiplex rheumatoid arthritis families suggests genetic overlap with other autoimmune diseases. Am J Hum Genet. 2001;68: 927–936. 11254450 10.1086/319518 PMC1275647

[48] Bowcock AM. The genetics of psoriasis and autoimmunity. Annu Rev Genomics Hum Genet. 2005;6: 93–122. 16124855 10.1146/annurev.genom.6.080604.162324

[49] Sawasaki T, Ogasawara T, Morishita R, Endo Y. A cell-free protein synthesis system for high-throughput proteomics. Proc Natl Acad Sci U S A. 2002;99: 14652–14657. 12409616 10.1073/pnas.232580399 PMC137474

[50] Matsumoto T, Hasegawa H, Onishi S, Ishizaki J, Suemori K, Yasukawa M. Protein kinase C inhibitor generates stable human tolerogenic dendritic cells. J Immunol. 2013;191: 2247–2257. 10.4049/jimmunol.1203053 23878315

[51] Matsuo S, Imai E, Horio M, Yasuda Y, Tomita K, Nitta K, et al Revised equations for estimated GFR from serum creatinine in Japan. Am J Kidney Dis. 2009;53: 982–992. 10.1053/j.ajkd.2008.12.034 19339088

[52] Duan L, Wang CY, Chen J, Gong Q, Zhu P, Zheng F, et al High-mobility group box 1 promotes early acute allograft rejection by enhancing IL-6-dependent Th17 alloreactive response. Lab Invest. 2011;91: 43–53. 10.1038/labinvest.2010.141 20714327

[53] Andrews BS, Eisenberg RA, Theofilopoulos AN, Izui S, Wilson CB, McConahey PJ, et al Spontaneous murine lupus-like syndromes: Clinical and immunopathological manifestations in several strains. J Exp Med. 1978;148: 1198–1215. 309911 10.1084/jem.148.5.1198 PMC2185049

[54] Pisetsky DS. DNA as a marker of cell death in systemic lupus erythematosus. Rheum Dis Clin North Am. 2004;30: 575–587. 15261342 10.1016/j.rdc.2004.04.009

[55] Xie C, Liang Z, Chang S, Mohan C. Use of a novel elution regimen reveals the dominance of polyreactive antinuclear autoantibodies in lupus kidneys. Arthritis Rheum. 2003;48: 2343–2352. 12905490 10.1002/art.11092

[56] Alexander JJ, Hack BK, Jacob A, Chang A, Haas M, Finberg RW et al Abnormal immune complex processing and spontaneous glomerulonephritis in complement factor H-deficient mice with human complement receptor 1 on erythrocytes. J Immunol. 2010;185: 3759–3767. 10.4049/jimmunol.1000683 20702729

[57] Grootscholten C, van Bruggen MC, van der Pijl JW, de Jong EM, Ligtenberg G, Derksen RH, et al Deposition of nucleosomal antigens (histones and DNA) in the epidermal basement membrane in human lupus nephritis. Arthritis Rheum. 2003;48: 1355–1362. 12746908 10.1002/art.10974

[58] Kalaaji M, Mortensen E, Jørgensen L, Olsen R, Rekvig OP. Nephritogenic lupus antibodies recognize glomerular basement membrane-associated chromatin fragments released from apoptotic intraglomerular cells. Am J Pathol. 2006;168: 1779–1792. 16723695 10.2353/ajpath.2006.051329 PMC1606630

[59] Alba P, Bento L, Cuadrado MJ, Karim Y, Tungekar MF, Abbs I et al Anti-dsDNA, anti-Sm antibodies, and the lupus anticoagulant: significant factors associated with lupus nephritis. Ann Rheum Dis. 2003;62: 556–560. 12759294 10.1136/ard.62.6.556 PMC1754557

[60] Tsirogianni A, Pipi E, Soufleros K. Relevance of anti-C1q autoantibodies to lupus nephritis. Ann N Y Acad Sci. 2009;1173: 243–251. 10.1111/j.1749-6632.2009.04750.x 19758158

[61] Trendelenburg M. Antibodies against C1q in patients with systemic lupus erythematosus. Springer Semin Immunopathol. 2005;27: 276–285. 16189648 10.1007/s00281-005-0007-y

[62] Bruns A, Bläss S, Hausdorf G, Burmester GR, Hiepe F. Nucleosomes are major T and B cell autoantigens in systemic lupus erythematosus. Arthritis Rheum. 2000;43: 2307–2315. 11037891 10.1002/1529-0131(200010)43:10<2307::AID-ANR19>3.0.CO;2-J

[63] Kalaaji M, Fenton KA, Mortensen ES, Olsen R, Sturfelt G, Alm P, et al Glomerular apoptotic nucleosomes are central target structures for nephritogenic antibodies in human SLE nephritis. Kidney Int. 2007;71: 664–672. 17332738 10.1038/sj.ki.5002133

[64] Nemoto K, Ito S, Yoshida C, Miyata M, Kojima M, Degawa M. Hepatic expression of spermatogenic genes and their transiently remarkable downregulations in Wistar-Kyoto rats in response to lead-nitrate administration: strain-difference in the gene expression patterns. J Toxicol Sci. 2011;36: 357–364. 21628963 10.2131/jts.36.357

[65] Caulfield JP, Farquhar MG. Distribution of annionic sites in glomerular basement membranes: their possible role in filtration and attachment. Proc Natl Acad Sci U S A. 1976;73: 1646–1650. 1064037 10.1073/pnas.73.5.1646 PMC430356

[66] Kanwar YS, Farquhar MG. Anionic sites in the glomerular basement membrane. In vivo and in vitro localization to the laminae rarae by cationic probes. J Cell Biol. 1979;81: 137–153. 90048 10.1083/jcb.81.1.137 PMC2111521

[67] Masutani K, Akahoshi M, Tsuruya K, Takumoto M, Ninomiya T, Kohsaka T, et al Predominance of Th1 immune response in diffuse proliferative lupus nephritis. Arthritis Rheum. 2001;44: 2097–2106. 11592372 10.1002/1529-0131(200109)44:9<2097::AID-ART360>3.0.CO;2-6

