# Supplementary figures and images for: Novel Autoantigens Associated with Lupus Nephritis
Source: PLoS One. 2015 Jun 22;10(6):e0126564. doi: 10.1371/journal.pone.0126564 (PMC4476694; doi:10.1371/journal.pone.0126564)

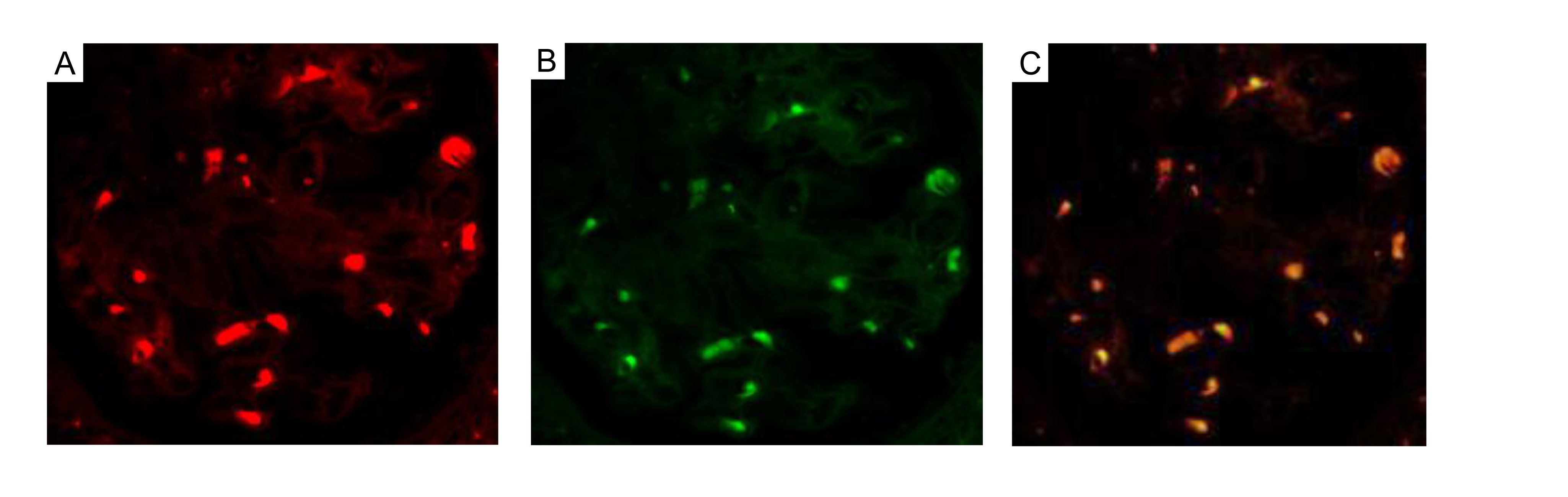

Supplement: S1 Fig — Formalin-fixed, paraffin-embedded sections of kidneys were stained with secondary antibody only; Alexa Fluor 546-conjugated anti-rabbit IgG antibody (A) or FITC-conjugated anti-rabbit IgG antibody (B). Panel (C) is merged image. The strong autofluorescence in each panel is mainly due to red blood cells. (TIF) [file pone.0126564.s001.tif]

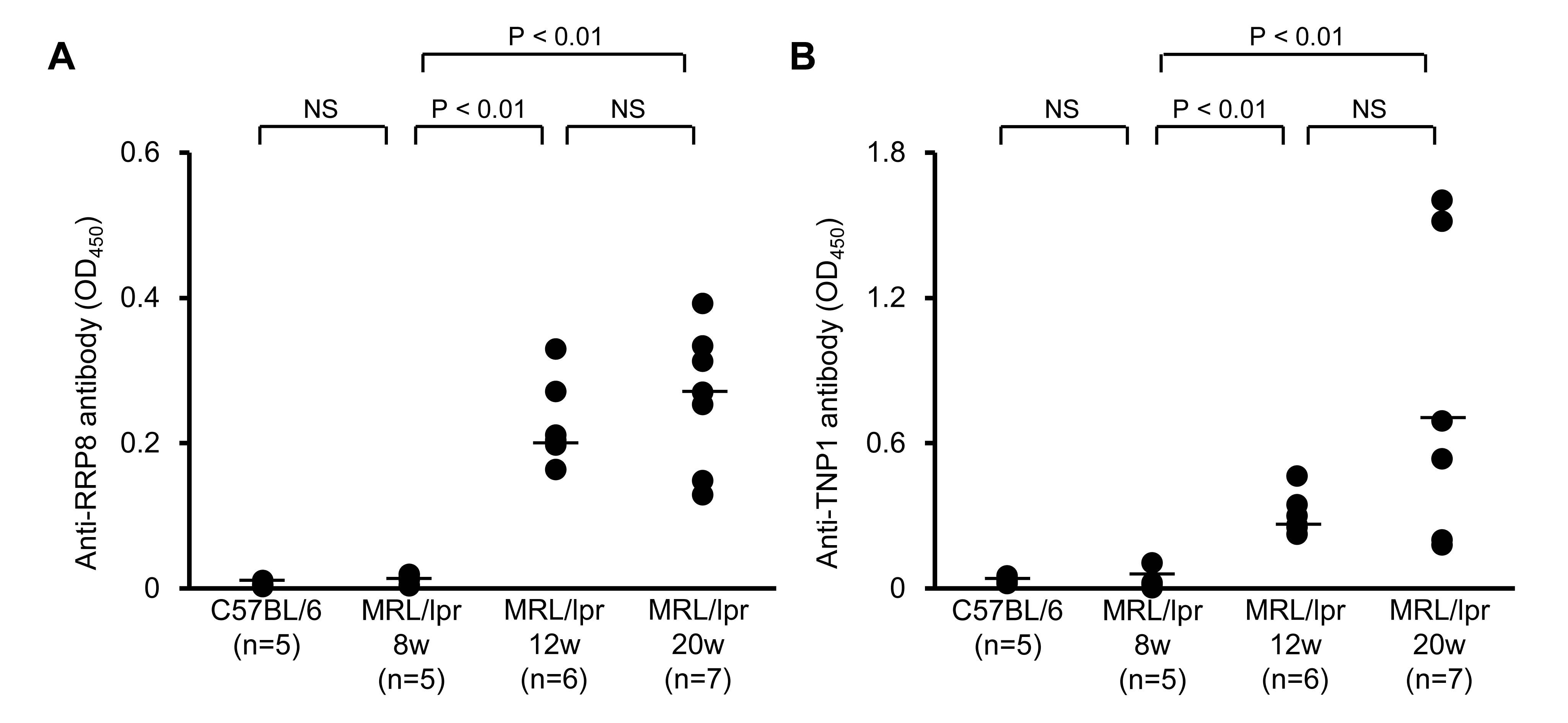

Supplement: S2 Fig — The serum samples were prepared from MRL/lpr mice at ages 8, 12, and 20 weeks during the development of nephritis. Serum samples were assayed by ELISA using purified mouse RRP8 or TNP1 protein. The sera of C57BL/6 mice were used as a control. (TIF) [file pone.0126564.s002.tif]

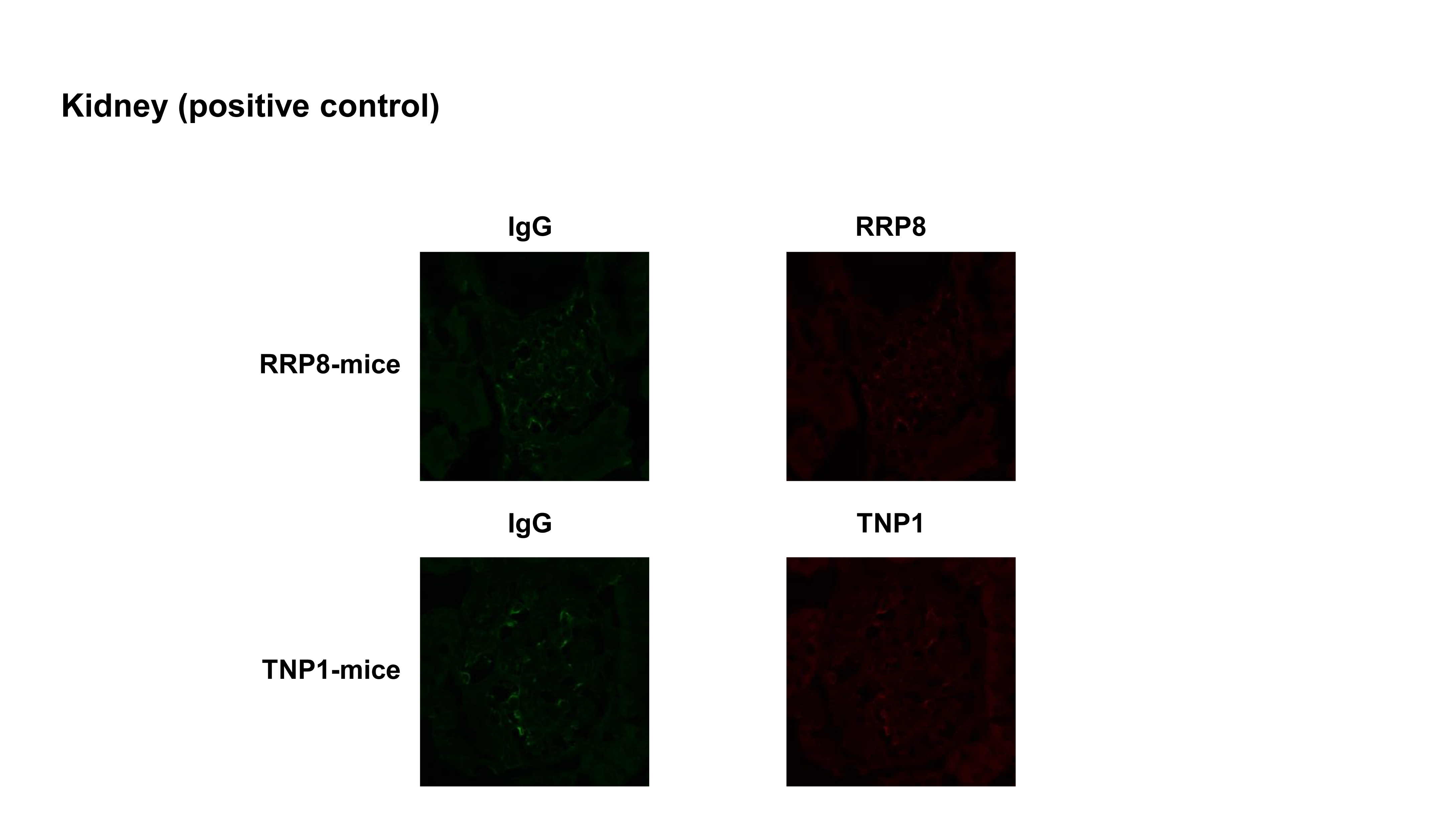

Supplement: S3 Fig — (TIF) [file pone.0126564.s003.tif]

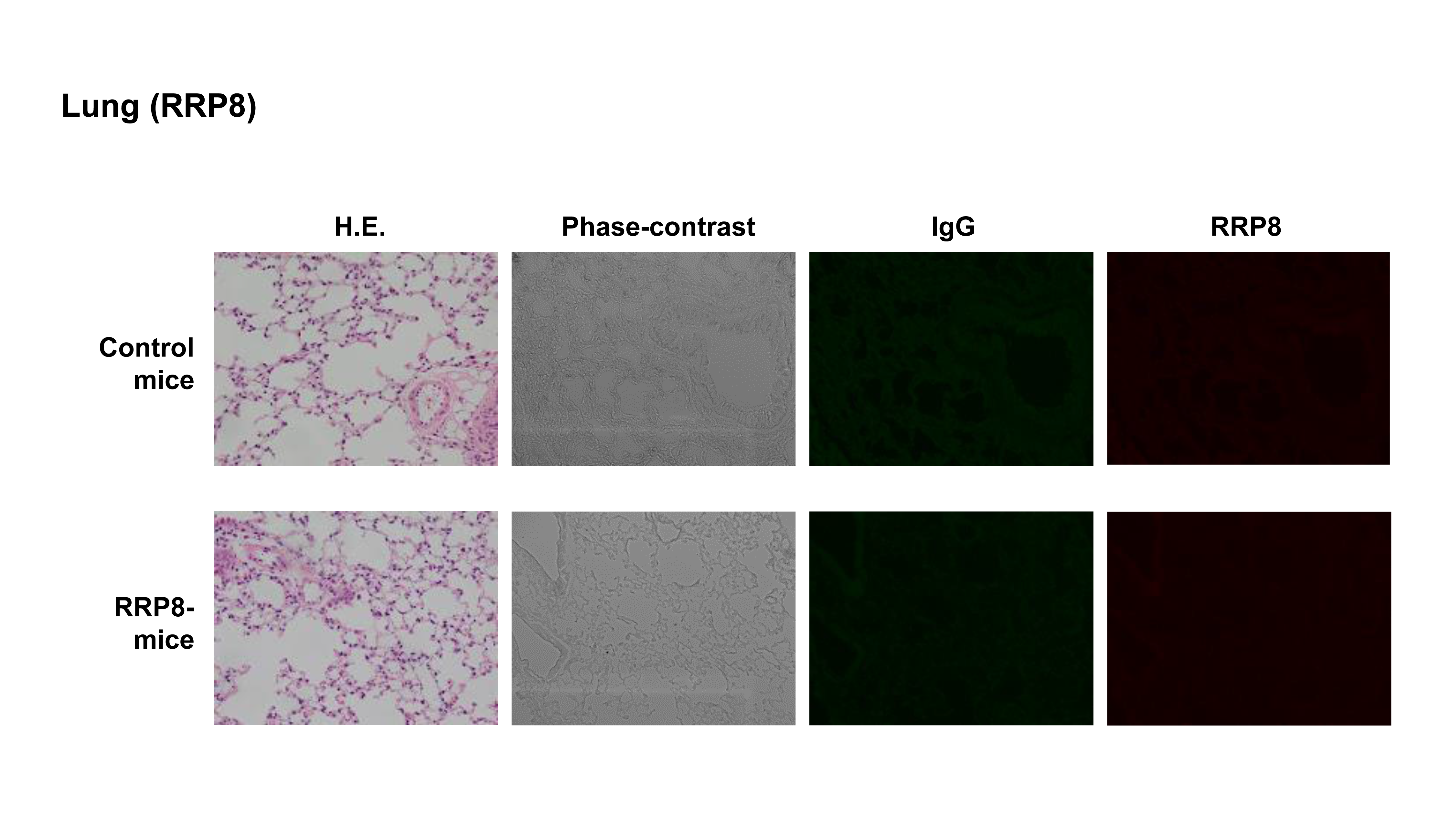

Supplement: S4 Fig — Normal C57BL/6 mice were used as a control. Representative photographs are shown. (TIF) [file pone.0126564.s004.tif]

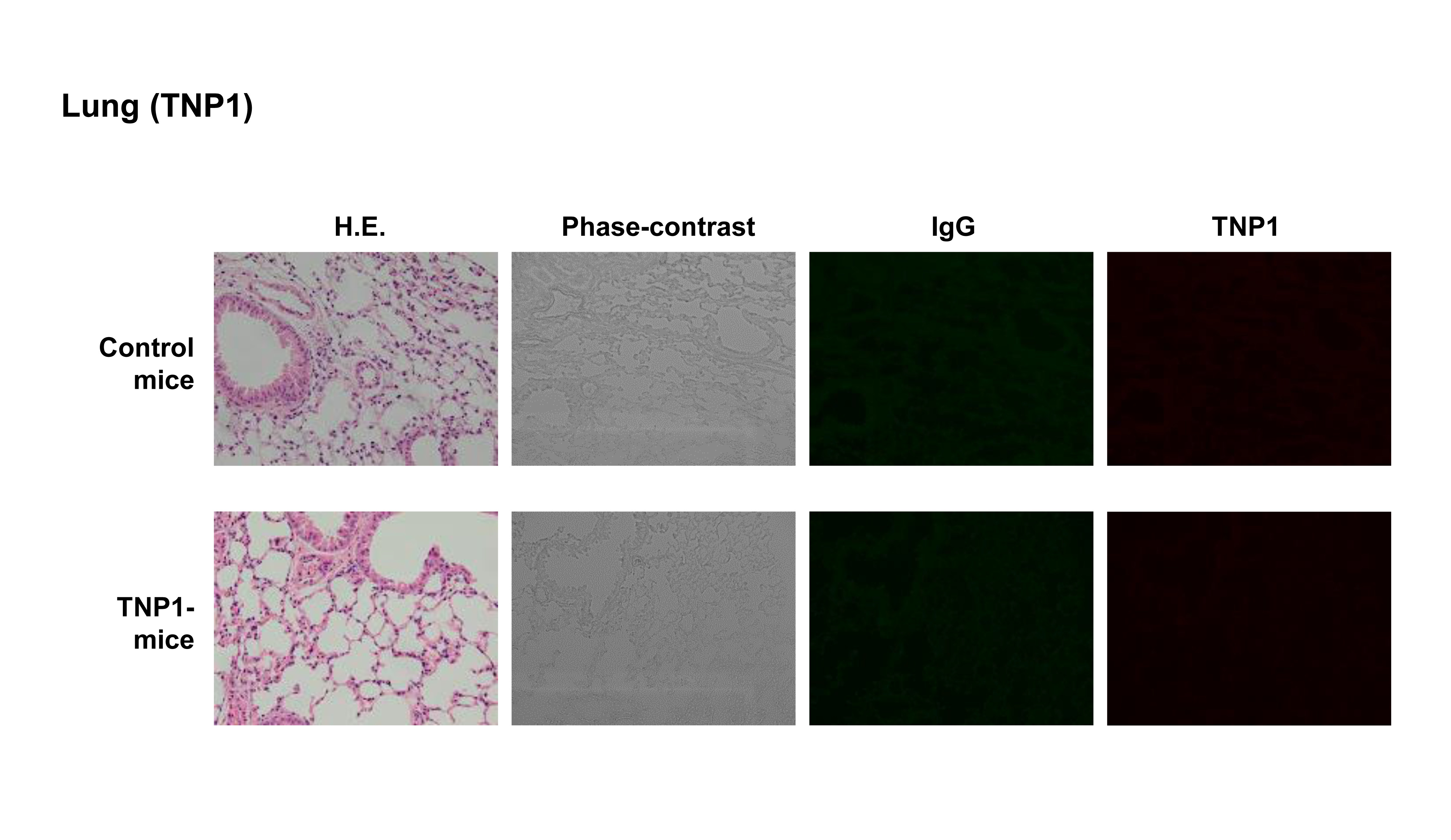

Supplement: S5 Fig — (TIF) [file pone.0126564.s005.tif]

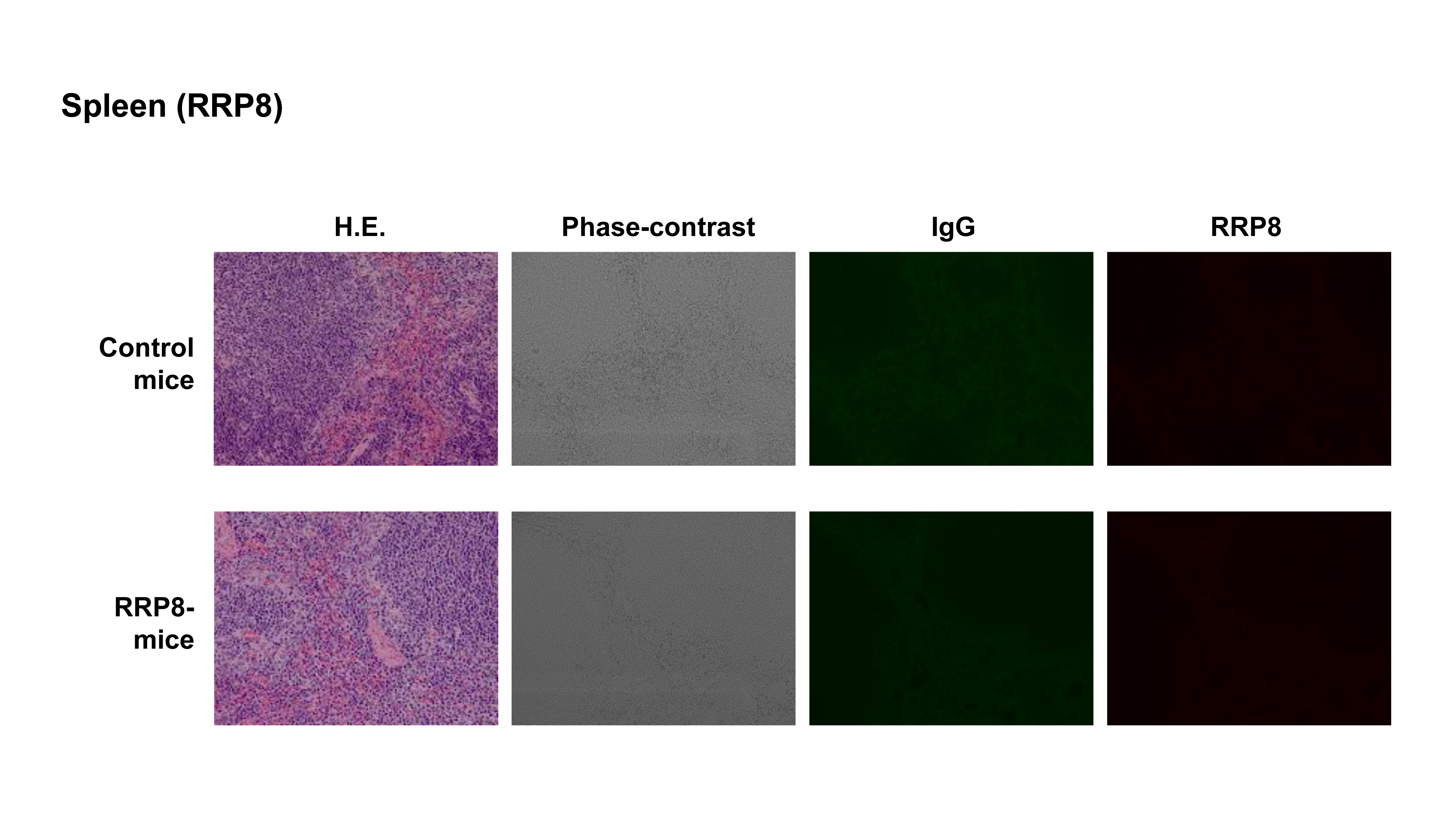

Supplement: S6 Fig — (TIF) [file pone.0126564.s006.tif]

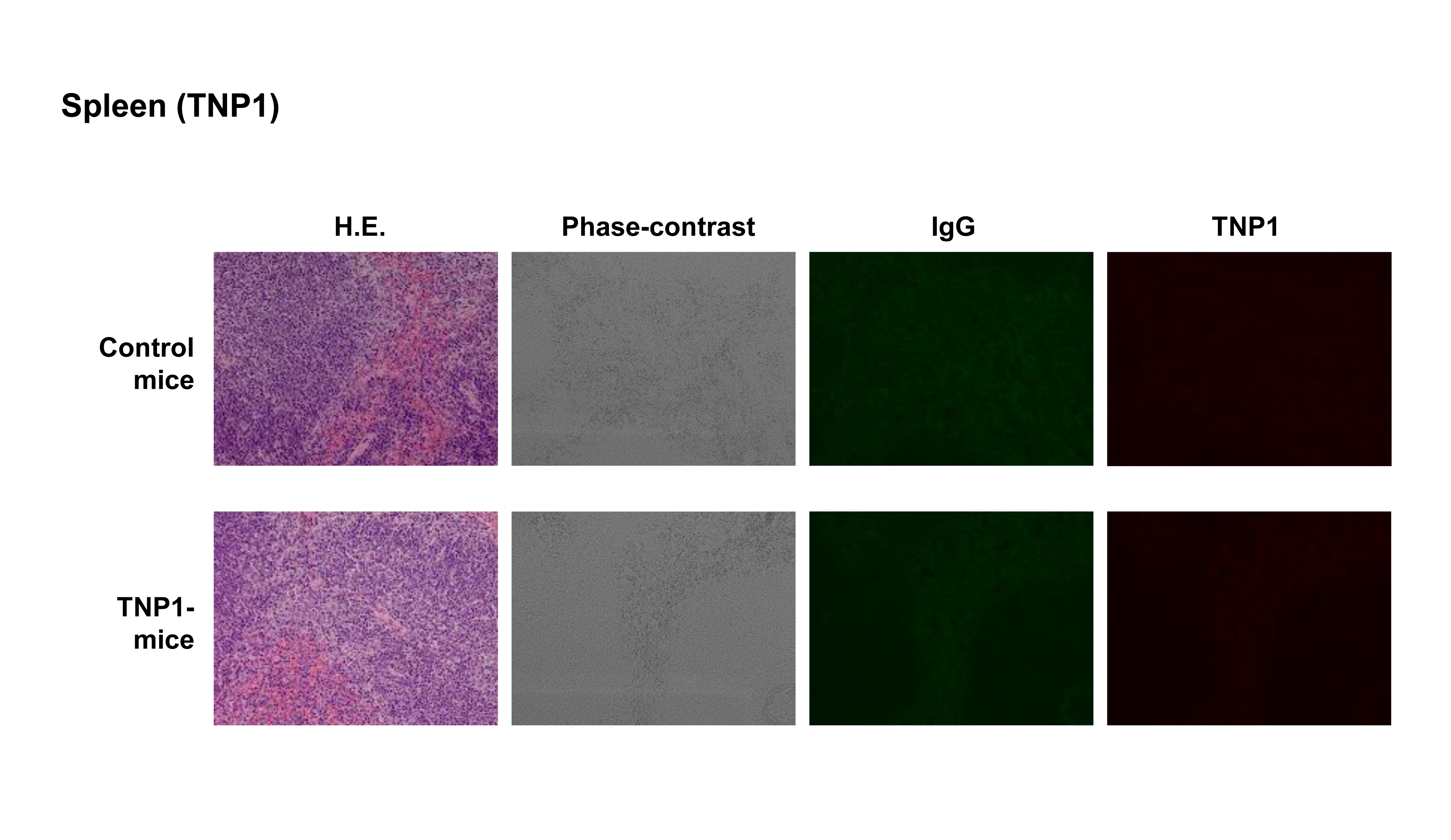

Supplement: S7 Fig — (TIF) [file pone.0126564.s007.tif]

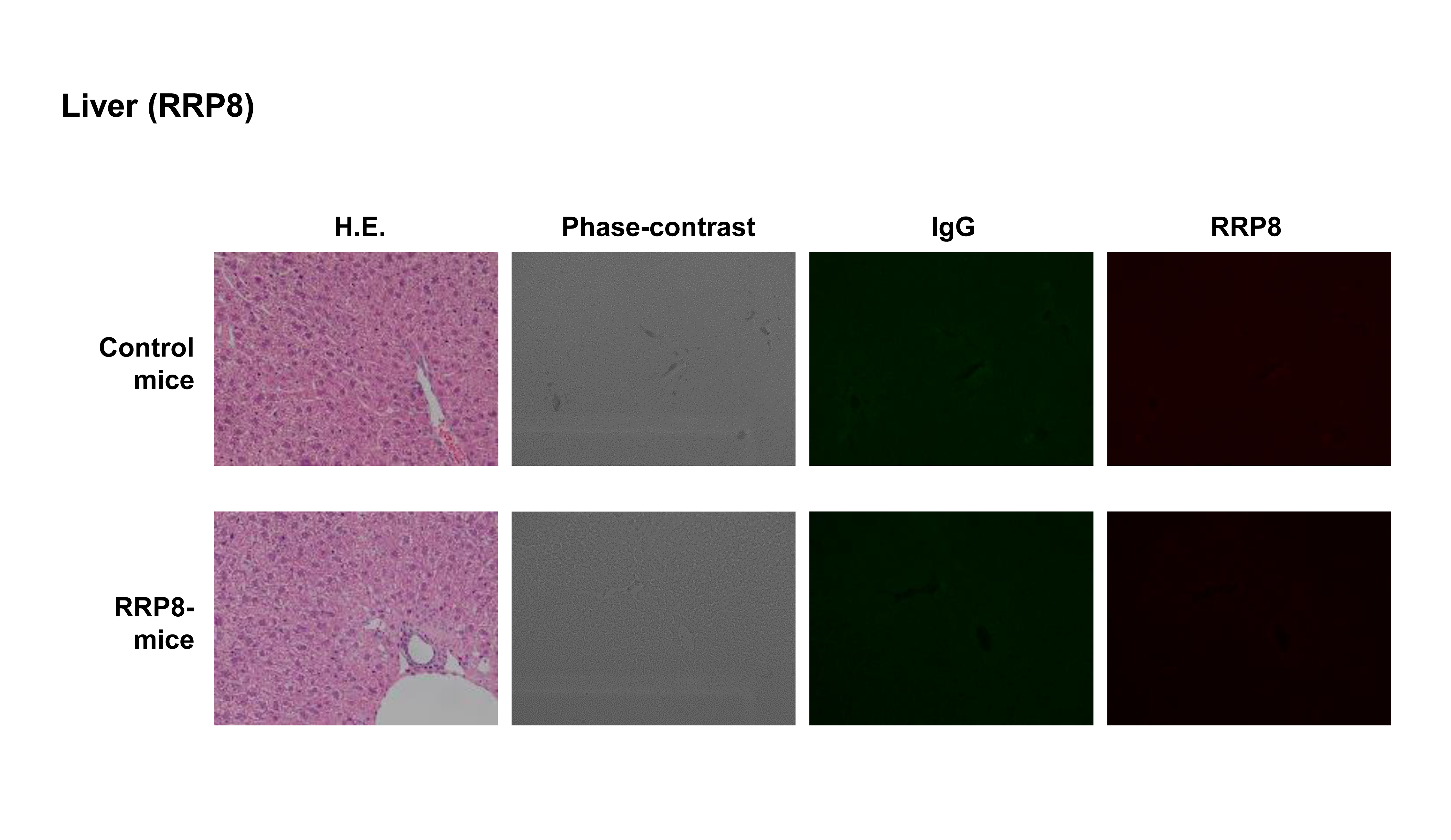

Supplement: S8 Fig — (TIF) [file pone.0126564.s008.tif]

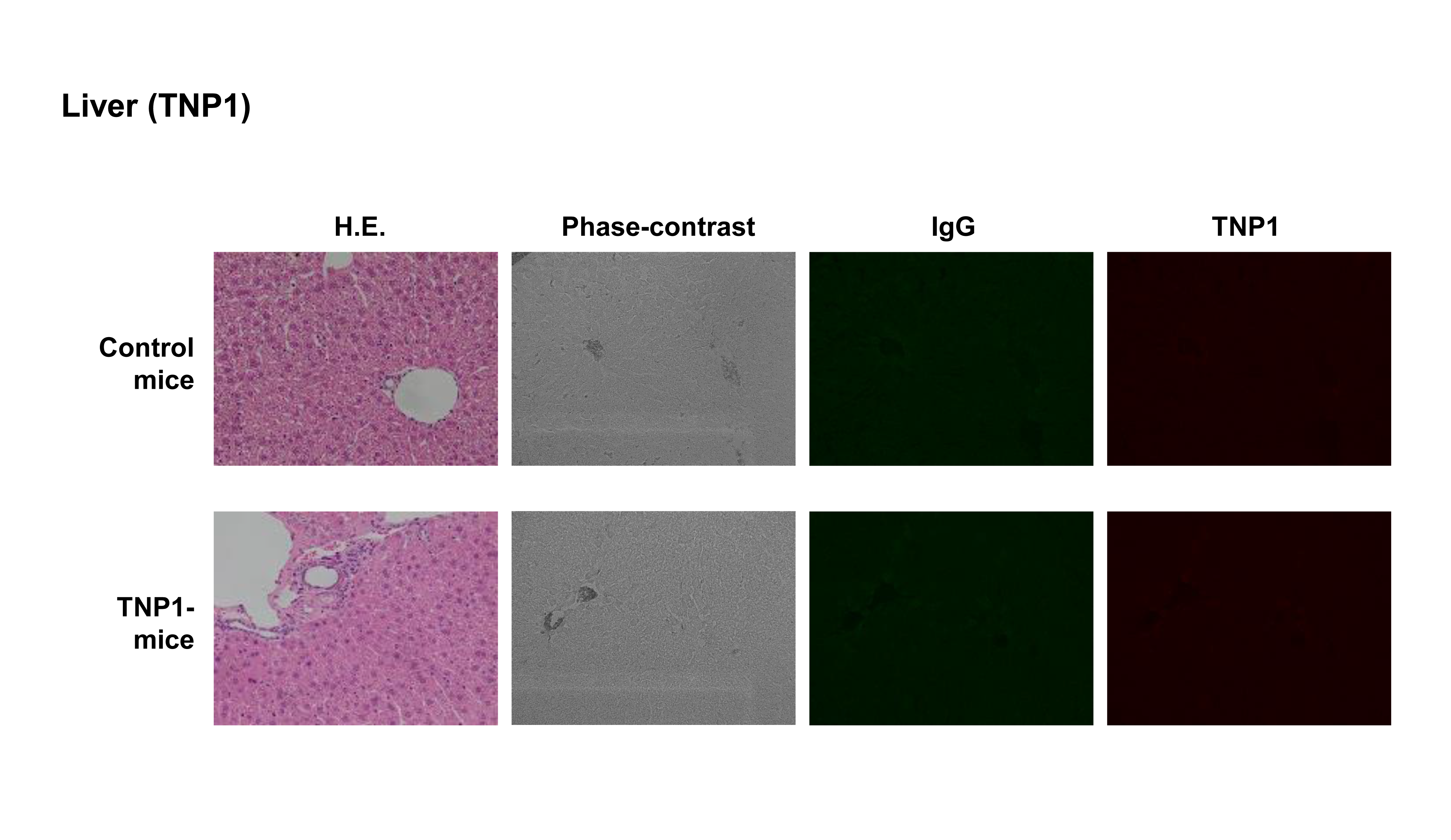

Supplement: S9 Fig — (TIF) [file pone.0126564.s009.tif]

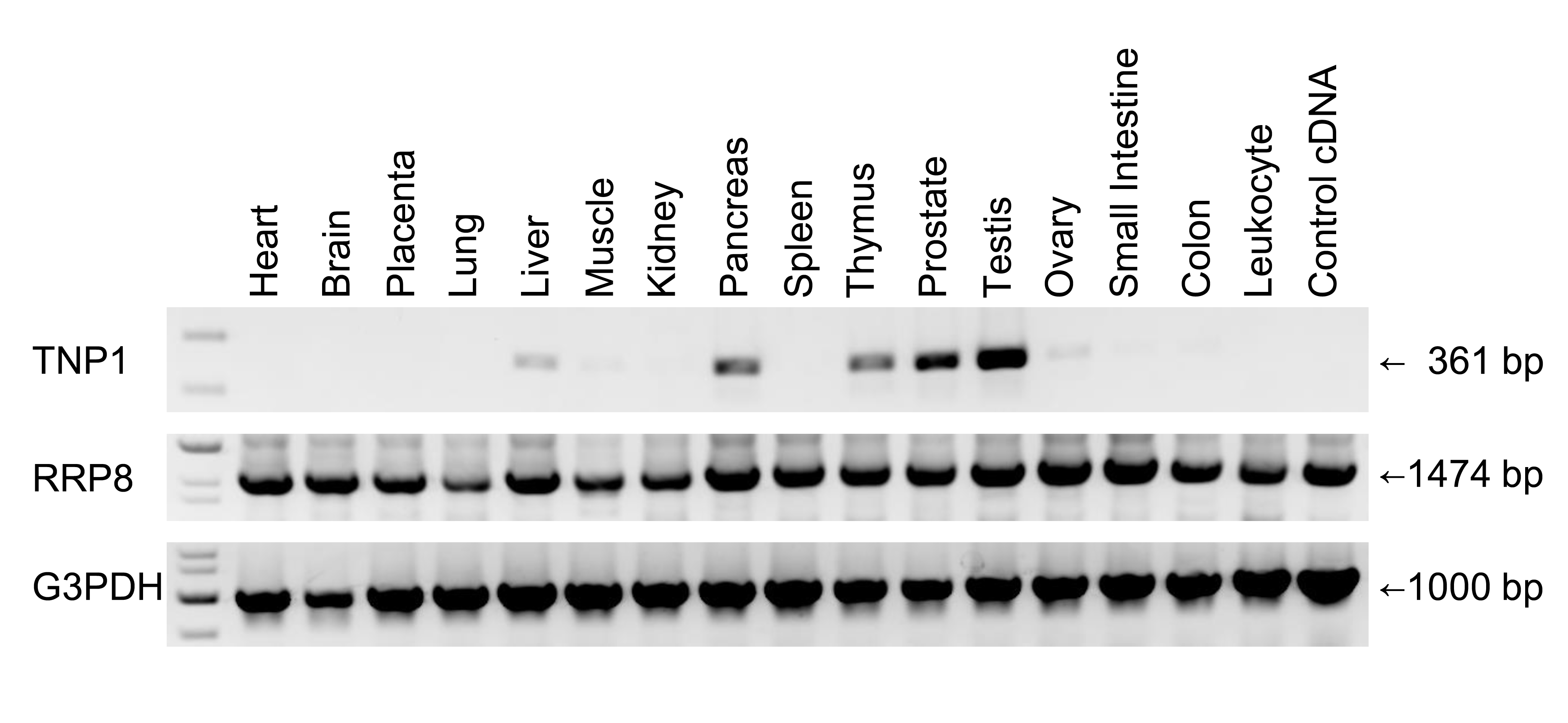

Supplement: S10 Fig — The expressions of TNP1 and RRP8 were analyzed with PCR using MTC cDNA panels. (TIF) [file pone.0126564.s010.tif]
