# Supplementary material for: Novel Autoantigens Associated with Lupus Nephritis
Source: PLoS One. 2015 Jun 22;10(6):e0126564. doi: 10.1371/journal.pone.0126564 (PMC4476694; doi:10.1371/journal.pone.0126564)
Supplement: S1 Table — (PDF) [file pone.0126564.s011.pdf]

Table S1. Clinical and laboratory data of 11 LN patients.

	LN1	LN2	LN3	LN4	LN5	LN6	LN7	LN8	LN9	LN10	LN11
Age (yr)	20	43	57	21	42	30	45	45	61	25	28
Sex	F	F	F	F	F	F	F	F	F	M	F
Disease duration (yr)	2	0.3	1	0.1	10	0.1	4	2	31	6	14
SLEDAI score	20	10	18	20	16	16	10	8	6	4	8
Antinuclear antibody#	1:640<br>sp	1:640<br>sp	1:2560<br>ho+sp	1:640<br>ho	1:640<br>ho+sp	1:2560<br>ho	1:10240<br>sp+cyto	1:2560<br>ho+sp	1:40<br>ho	1:160<br>ho+sp	1:640<br>ho+sp
Anti-dsDNA antibodies (IU/ml)<br>(normal range <12)	66	25	65	194	378	710	49	90	5.9	2.4	59
Other positive antibodies*1	anti-Sm	anti-Sm<br>anti-β2 GPI<br>aCL-IgG	anti-SSA<br>anti-SSB	anti-SSA<br>aCL-IgG	anti-Sm<br>anti-SSA<br>anti-β2 GPI<br>aCL-IgG	anti-SSA	anti-Sm<br>aCL-IgG	anti-Sm<br>anti-β2 GPI<br>aCL-IgG	anti-SSA	anti-Sm<br>aCL-IgG<br>anti-β2GPI	anti-Sm<br>anti-SSA
C3 (mg/dl)<br>(normal range 65-135)	23	38	22	24	22	20	50	41	60	108	60
C4 (mg/dl)<br>(normal range 13-35)	2.5	4.4	2.5	4.1	3.7	2.7	4.2	6	11	23	8.9
WBC (/μl)	7700	2500	2400	1300	4600	3900	3700	5300	6200	9600	6500
Platelet (×10^4/μl)	8.2	23.4	10.7	24.5	15.3	16.2	21.3	28.5	18.6	31.5	34.9
Urine protein (g/day)	1.1	0.7	1.98	2.4	1.3	2.18	3.59	0.5	1.45	1.1	0.84
Urine sedimentation	n.a.d.	n.a.d.	RBC 10/HPF	RBC 10/HPF	WBC 40/HPF<br>Waxy casts<br>WBC casts	RBC 100/HPF	n.a.d.	n.a.d.	n.a.d.	n.a.d.	RBC 7.4/HPF
CRP (mg/dl)	0.43	0.05	0.53	0.35	0.18	0.08	0.29	0.05	0.18	0.03	0.06
Creatinine (mg/dl)	0.47	0.51	0.8	0.47	0.75	0.6	0.53	0.5	0.46	1.43	0.56
Pathologic classification	III(A)+V	III(A)	IV-G(A)+V	II	III(A)+V	III(A/C)	V	n.e.	n.e.	n.e.	n.e.
Manifestations	Fever,<br>Depression,<br>Livedo reticularis	Fever	Arthritis,<br>Pericarditis	Fever,<br>Rash,<br>Alopecia,<br>Arthralgia	Oral ulcers,<br>Malar rash	Oral ulcers,<br>Arthritis	Pericarditis,<br>ILD	(past)<br>Malar rash,<br>Arthritis	(past)<br>Arthritis,<br>anti-dsDNA	(past)<br>Arthritis,<br>Neutropenia,<br>anti-dsDNA	(past)<br>Rash,<br>Arthritis

ho; homogeneous, sp; speckled, cyto; cytoplasmic

*1 We examined the following autoantibodies; Sm, SSA, SSB, cardiolipin (CL) and β2 glycoprotein I (GPI).

LN; lupus nephritis, ILD; interstitial lung disease, n.a.d.; no abnormality detected, n.e.: not examined
