# Supplementary material for: Novel Autoantigens Associated with Lupus Nephritis
Source: PLoS One. 2015 Jun 22;10(6):e0126564. doi: 10.1371/journal.pone.0126564 (PMC4476694; doi:10.1371/journal.pone.0126564)
Supplement: S2 Table — (PDF) [file pone.0126564.s012.pdf]

Table S2. Clinical and laboratory data of patient A and B.

	Patient A (LN2)		Patient B (SLE3)	
	active	inactive	active	inactive
Age (yr)	43	44	25	26
Disease duration (yr)	0.3	0.8	0.3	1
SLEDAI score	10	4	14	4
Anti-dsDNA antibodies (IU/ml) (normal range <12)	25	49	495	51
C3 (mg/dl) (normal range 65-135)	38	46	43	69
C4 (mg/dl) (normal range 13-35)	4.4	6.4	<1.7	3.1
WBC (/μl)	2500	5900	2300	5300
Platelet ($\times 10^4$/μl)	23.4	22.5	13.2	17.5
Urine protein (g/day)	0.7	0	0	0.01
Urine sedimentation	n.a.d.	n.a.d.	n.a.d.	n.a.d.
CRP (mg/dl)	0.05	0.05	0.29	0.01
Creatinine (mg/dl)	0.51	0.55	0.45	0.62
Manifestations	Fever<br>Nephritis<br>(Class IIIA)		Fever<br>Arthritis<br>Pericarditis<br>Pleurisy	

Patient A (LN2); Nephritis (Class III(A)), Patient B (SLE3); Serositis (without nephritis).
n.a.d.; no abnormality detected.
