# Supplementary material for: Novel Autoantigens Associated with Lupus Nephritis
Source: PLoS One. 2015 Jun 22;10(6):e0126564. doi: 10.1371/journal.pone.0126564 (PMC4476694; doi:10.1371/journal.pone.0126564)
Supplement: S3 Table — (PDF) [file pone.0126564.s013.pdf]

Table S3. Clinical and laboratory data of 20 patients analyzed with immunoprecipitation.

	SLE1	SLE2	SLE3	SLE4	SLE5
Age (yr)	39	41	25	40	22
Sex	F	F	F	F	F
Disease duration (yr)	0.1	0.1	0.3	10	11
SLEDAI score	12	16	14	4	2
Antinuclear antibody#	1:2560 ho	1:10240 ho	1:10240 sp	1:2560 sp	1:2560 ho
Anti-dsDNA antibodies (IU/ml) (normal range <12)	667	179	495	30	8.2
Other positive antibodies*1	anti-SSA anti-SSB aCL-IgG	anti-SSA anti-SSB	anti-Sm anti-SSA anti-SSB	anti-SSA anti-β2 GPI aCL-IgG	anti-β2 GPI aCL-IgG
C3 (mg/dl) (normal range 65-135)	16	55	43	67	79
C4 (mg/dl) (normal range 13-35)	1.8	28	<1.7	4.5	5.9
WBC (/μl)	2300	2700	2300	3800	6600
Platelet (×10^4/μl)	16.3	24.5	13.2	19.5	22.8
Urine protein (g/day)	0.15	0	0	0.095	0
Urine sedimentation	RBC 4.5/HPF WBC 1.7/HPF	n.a.d.	n.a.d.	n.a.d.	n.a.d.
CRP (mg/dl)	0.03	0.71	0.29	0.1	0.04
Creatinine (mg/dl)	0.68	0.44	0.45	0.42	0.55
Manifestations	Fever, Malar rash, Chilblain lupus, Arthritis	Fever, Malar rash, Psychosis	Fever, Arthritis, Pericarditis, Pleurisy	Lymphopenia, Photosensitivity	Hemolytic anemia, Photosensitivity

	DM/PM1	DM/PM2	DM/PM3	DM/PM4	DM/PM5
Age (yr)	30	31	65	63	60
Sex	F	F	F	M	F
Disease duration (yr)	0.3	0.3	0.2	0.5	24
CK (IU/l)	3212	10635	1949	4049	876
Antinuclear antibody	negative	negative	negative	negative	negative
Positive autoantibodies*2	anti-Jo1 anti-SSA	anti-SRP anti-SSA	anti-Jo1	n.a.d.	anti-Jo1
Manifestations	Gottron's sign, ILD	Gottron's papules	Gottron's sign, Cuticular overgrowth, ILD	Gottron's sign, Shawl sign, ILD	Gottron's papules, Periungual erythema, Calcinosis cutis, ILD

	SSc1	SSc2	SSc3	SSc4	SSc5
Age (yr)	69	73	61	63	54
Sex	F	F	F	F	F
Disease duration (yr)	12	4	0.4	3	9
Antinuclear antibody#	1:2560 ho	1:160 ho	1:640 ho	1:1280 dis-sp	1:40 ho+cyto
Positive autoantibodies*3	anti-Scl70	anti-Scl70	anti-Scl70	anti-centromere	n.a.d.
Skin thickening	face, forearms, upper arms, hands, thighs, trunk	hands, forearms	hands, forearms	face, hands, forearms	hands, forearms
Manifestations	Calcinosis cutis, ILD, CIPO	ILD	ILD		ILD, PH

Five LN patients in this experiment are LN1 to LN5 (shown in Table S1).

ho; homogeneous, sp; speckled, dis-sp; discrete-speckled, cyto; cytoplasmic

*1 We examined the following autoantibodies; Sm, SSA, SSB, cardiolipin (CL) and β2 glycoprotein I (GPI).

*2 We examined the following autoantibodies; SSA, Jo1, M2 (PDC-E2, BCOADC-E2, OGDC-E2), PM-Scl-100, PL-7, PL-12, Mi-2, Ku (p70/80), SRP and Rib-P.

*3 We examined anti-Scl70 and anti-centromere antibodies.

LN; lupus nephritis, SLE; systemic lupus erythematosus, DM/PM; dermatomyositis / polymyositis, SSc; systemic sclerosis. ILD; interstitial lung disease, CIPO; chronic intestinal pseudo-obstruction, PH; pulmonary hypertension, n.a.d.; no abnormality detected.
