# Supplementary material for: Novel Autoantigens Associated with Lupus Nephritis
Source: PLoS One. 2015 Jun 22;10(6):e0126564. doi: 10.1371/journal.pone.0126564 (PMC4476694; doi:10.1371/journal.pone.0126564)
Supplement: S4 Table — (PDF) [file pone.0126564.s014.pdf]

Table S4. Information on 238 patients and 41 healthy individuals analyzed with ELISA.

	n	Female ratio (%)	Age (yr)	Disease duration (yr)
Lupus nephritis	11	90.9	37.9 ± 14.1	6.4 ± 9.3
SLE without nephritis	64	90.6	43.7 ± 17.1	10.7 ± 8.3
Dermatomyositis / polymyositis	30	86.7	57.6 ± 16.2	8.9 ± 9.7
Systemic sclerosis	16	100	58.9 ± 10.4	8.7 ± 6.3
Mixed connective tissue Disease	15	93.3	45.5 ± 12.3	7.3 ± 4.9
Rheumatoid arthritis	55	69.1	63.8 ± 13.9	7.8 ± 9.5
Sjögren syndrome	12	100	50.6 ± 16.9	7.3 ± 5.8
Behçet's disease	22	31.8	48.1 ± 12.1	7.9 ± 5.5
ANCA-associated vasculitis	13	53.8	64.5 ± 13.3	4.3 ± 2.6
Healthy controls	41	68.3	35.4 ± 17.1	

SLE; systemic lupus erythematosus, ANCA; anti-neutrophil cytoplasmic antibody.
